# The Abi-domain Protein Abx1 Interacts with the CovS Histidine Kinase to Control Virulence Gene Expression in Group B *Streptococcus*

**DOI:** 10.1371/journal.ppat.1003179

**Published:** 2013-02-21

**Authors:** Arnaud Firon, Asmaa Tazi, Violette Da Cunha, Sophie Brinster, Elisabeth Sauvage, Shaynoor Dramsi, Douglas T. Golenbock, Philippe Glaser, Claire Poyart, Patrick Trieu-Cuot

**Affiliations:** 1 Institut Pasteur, Unité de Biologie des Bactéries Pathogènes à Gram-Positif, Paris, France; 2 Centre National de la Recherche Scientifique, CNRS ERL3526, Paris, France; 3 Institut Cochin, Université Sorbonne Paris Descartes, Paris, France; 4 Institut National de la Santé et de la Recherche Médicale, INSERM U1016, Paris, France; 5 Centre National de la Recherche Scientifique, CNRS UMR8104, Paris, France; 6 Assistance Publique Hôpitaux de Paris, Service de Bactériologie, Centre National de Référence des Streptocoques, Hôpitaux Universitaires Paris Centre Cochin-Hôtel Dieu-Broca, Paris, France; 7 Centre National de la Recherche Scientifique, CNRS UMR3525, Paris, France; 8 University of Massachusetts Medical School, Division of Infectious Diseases and Immunology, Department of Medicine, Worcester, Massachusetts, United States of America; University of Birmingham, United Kingdom

## Abstract

Group B *Streptococcus* (GBS), a common commensal of the female genital tract, is the leading cause of invasive infections in neonates. Expression of major GBS virulence factors, such as the hemolysin operon *cyl*, is regulated directly at the transcriptional level by the CovSR two-component system. Using a random genetic approach, we identified a multi-spanning transmembrane protein, Abx1, essential for the production of the GBS hemolysin. Despite its similarity to eukaryotic CaaX proteases, the Abx1 function is not involved in a post-translational modification of the GBS hemolysin. Instead, we demonstrate that Abx1 regulates transcription of several virulence genes, including those comprising the hemolysin operon, by a CovSR-dependent mechanism. By combining genetic analyses, transcriptome profiling, and site-directed mutagenesis, we showed that Abx1 is a regulator of the histidine kinase CovS. Overexpression of Abx1 is sufficient to activate virulence gene expression through CovS, overcoming the need for an additional signal. Conversely, the absence of Abx1 has the opposite effect on virulence gene expression consistent with CovS locked in a kinase-competent state. Using a bacterial two-hybrid system, direct interaction between Abx1 and CovS was mapped specifically to CovS domains involved in signal processing. We demonstrate that the CovSR two-component system is the core of a signaling pathway integrating the regulation of CovS by Abx1 in addition to the regulation of CovR by the serine/threonine kinase Stk1. In conclusion, our study reports a regulatory function for Abx1, a member of a large protein family with a characteristic Abi-domain, which forms a signaling complex with the histidine kinase CovS in GBS.

## Author Summary

The gram-positive *Streptococcus* genus includes three major human pathogens that are members of the normal microflora: *Streptococcus pneumoniae* (also known as the pneumococcus), *Streptococcus pyogenes* (Group A *Streptococcus*), and *Streptococcus agalactiae* (Group B *Streptococcus*). Their carriage in the population is highly dynamic and mostly asymptomatic. However, each of these species can cause a wide spectrum of diseases, from local infections to systemic and fatal infections including septicemia and meningitis. Expression of streptococcal virulence-associated genes is tightly regulated at the transcriptional level. However, the signal(s) and the precise molecular events controlling the switch from commensalism to virulence are not yet understood. In this study, we identified and characterized a bacterial protein essential for virulence gene expression in Group B *Streptococcus*, the main pathogen of neonates. We show that this transmembrane protein, named Abx1, interacts with the histidine kinase CovS to modulate the activity of the major regulator of virulence CovR. We define how a core set of four proteins, Abx1, CovS, CovR, and the serine/threonine kinase Stk1, interact to control the expression of virulence genes in *S. agalactiae*. We propose that Abx1-like proteins, that are widespread in bacteria, might be part of a conserved mechanism of two-component system regulation.

## Introduction

Some commensal microorganisms are also opportunistic pathogens. Harmless, and potentially beneficial, they may become causative agents of local or systemic infections [1], [2]. To date, the signals dictating the switch from commensalism to virulence are mainly unknown. The complex set of genetic and environmental factors thought to be involved would affect the equilibrium between the host and the microbes. Deciphering the molecular events that govern the transition between commensalism and virulence will contribute to understanding and controlling infections due to opportunistic pathogens.

*Streptococcus agalactiae* (Group B *S* *treptococcus*, GBS) is a commensal bacterium of the adult gastro-intestinal tract and is present asymptomatically in the vaginal flora of 10–30% of healthy women [3]. However, GBS is the leading cause of invasive infections in neonates (pneumonia, septicaemia, and meningitis) and a serious cause of mortality or morbidity in adults with underlying diseases [4]–[6]. As with most streptococci, the ability of GBS to cause infections is multifactorial [7]. The main virulence-associated GBS proteins identified to date are secreted or surface components, including the ß-hemolysin/cytolysin and specific adhesins [7]–[10].

Expression of several major GBS virulence genes is regulated at the transcriptional level by a two-component system (TCS) called CovSR (Control of virulence Sensor and Regulator; also known as CsrSR) [10]–[13]. TCS is the main signaling mechanism used by bacteria to respond to their changing environments [14]. CovSR controls the GBS response to acid stress *in vitro* and is necessary *in vivo* at several steps of the infectious process, such as resistance to macrophage killing and penetration of the blood-brain barrier [15]–[18]. Depending on the model of infection, inactivation of the CovSR system leads to a decrease or an increase of virulence [11]–[13], [15], suggesting that this system is tightly regulated in space and time to specifically adapt the bacterial virulence capacities to the infected compartments of the host. CovSR orthologs are present in several streptococcal species, including *Streptococcus pyogenes* (Group A Streptococcus, GAS), where their function as a master regulator of virulence gene expression is conserved [19]–[23].

The CovSR system belongs to the EnvZ/OmpR family of TCS, where CovS is the membrane-bound histidine protein kinase (HK) and CovR is the cytosolic response regulator (RR). Typically, HK is the sensor of an environmental stimulus that induces its autophosphorylation and, subsequently, phosphorylation of its cognate RR [14]. Phosphorylation of the RR alters its function, generally by modifying its binding affinity for target promoter regions and thereby changing expression of specific genes or operons. In contrast to the majority of TCS, the streptococcal CovSR regulatory pathway is a combination of two antagonistic negative regulators [24]. Indeed, in both GBS and GAS, the phosphorylated CovR acts mainly as a transcriptional inhibitor by direct binding to target gene promoters [11], [13], [25]–[27]. It was therefore suggested, but not formally demonstrated *in vitro*, that CovS acts mainly as a phosphatase on CovR to de-repress virulence gene expression during infection [13], [24], [28]. Currently, it is not known whether CovS is activated by general stresses [16], [28] and/or is a direct sensor of specific ligands [22]. The latter hypothesis is supported in GAS by the identification of extracellular Mg^2+^ and sub-inhibitory concentrations of LL-37, an antimicrobial peptide secreted by innate immune cells, as specific ligands that respectively activate and inhibit CovS activity [22], [29], [30].

Additional levels of TCS regulation exist that do not depend directly on environmental signal sensing by the HK sensor [31]–[33]. These cellular regulators target either the HK or the RR and were named “third components”, “auxiliary proteins”, “adaptors” or “TCS connectors”. While keeping the specific molecular links between an HK and its cognate RR, the additional cellular regulator allows to coordinate the cellular response and/or to integrate multiple signals [33]. This is the case in GBS where CovR activity is modulated by a second signaling pathway mediated by the serine/threonine kinase Stk1 [15], [25], [34]. Direct phosphorylation of the CovR T_65_ threonyl residue by Stk1 decreases CovR activity and interferes negatively with CovS-dependent phosphorylation of the D_53_ aspartyl residue [25]. These CovS- and Stk1- signaling pathways converge on CovR and are both necessary for GBS virulence [15].

In this study, we identified and characterized a transmembrane protein, Abx1, belonging to a large family of bacterial proteins of unknown function that have a conserved domain called Abi [35], [36]. We provide evidence that the putative protease activity of Abx1, inferred from its similarity to eukaryotic CaaX proteases [35], [36], is not necessary for its function. Instead, we demonstrate that Abx1 is an additional partner of the CovSR system that is necessary to regulate CovS activity by a protein-protein interaction. We show that the Abx1-CovS signaling complex and the Stk1-dependent pathway are both necessary to control CovR activity. In addition to defining the genetic network controlling virulence gene expression in GBS, we provide the first report of a cellular regulator of HK activity belonging to the large family of bacterial Abi-domain proteins.

## Results

### Gbs1532/Abx1 is required for pigment production and hemolysis in GBS

The ß-hemolysin/cytolysin expressed by GBS is an important virulence factor encoded within a cluster of twelve genes forming the *cyl* operon [8], [37], [38]. Hemolytic activity of GBS is always associated with the synthesis of an orange pigment. Among the 12 genes of the *cyl* operon (*gbs0644-gbs0655* genes), the CylE protein (*gbs0651*) is the critical determinant for the dual hemolytic/pigmentation phenotypes [8], [38]–[40]. Inactivation of *cylE* abolishes hemolytic activity and pigmentation, and the mutant is less virulent in animal models of systemic infections [39]–[41].

**Figure 1:**
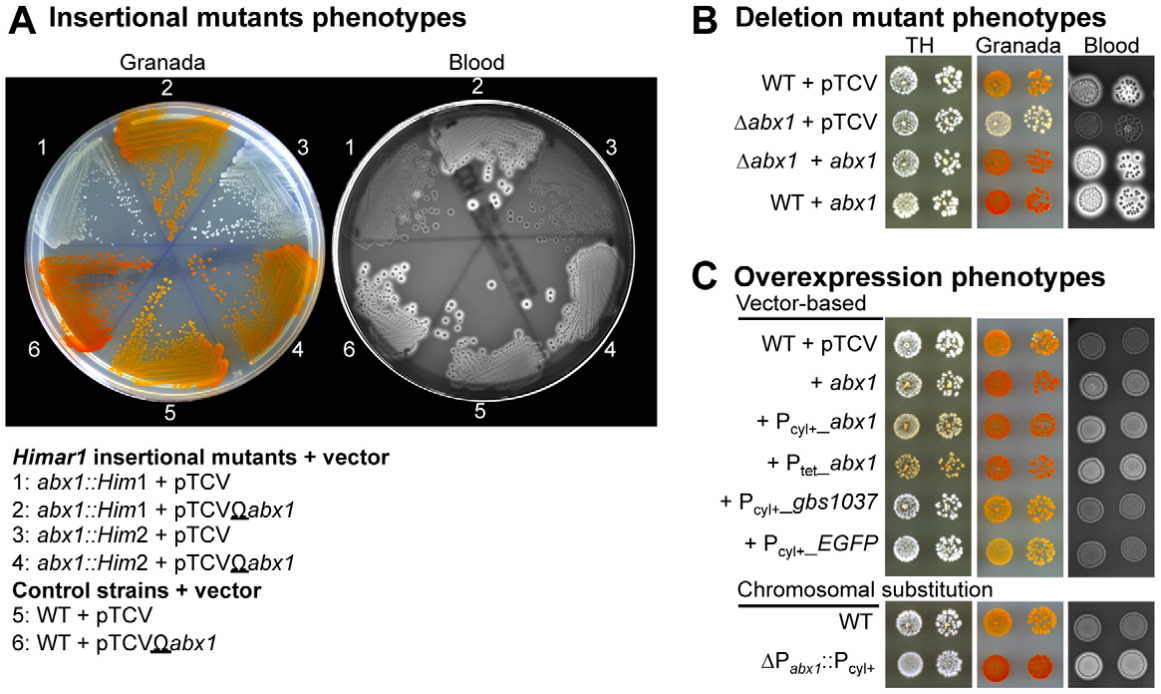
The Abx1 (Gbs1532) protein is essential for hemolytic activity and pigment production in GBS. (A) ß-hemolytic and pigmentation phenotypes of NEM316 wild-type strain (WT) and of two independent insertional mutants (*abx1::Him*1 and *abx1::Him*2) obtained by random transposon mutagenesis. Strains contained the empty vector pTCV or the complementing vector pTCVΩ*abx1* where *abx1* is transcribed from its own promoter. Pigment production was assayed on Granada agar plates and hemolytic activities on Columbia horse blood (5%) agar plates. The clear halos surrounding bacterial colonies (black dots) on blood plates correspond to lysed erythrocytes. Plates are supplemented with erythromycin (10 µg/ml) for plasmid maintenance. (B) Phenotypes of in-frame *abx1* deletion mutant (Δ*abx1*). Strains contained the same vectors as in (A). Overnight cultures in TH were diluted in fresh media and approximately 10^3^ and 10^2^ CFU were spotted on TH, Granada, and Columbia horse blood agar plates plus erythromycin. Pictures were taken after 24–36 h of growth at 37°C. (C) Hemolytic and pigmentation phenotypes associated with *abx1* overexpression. WT strain with the empty vector pTCV, the complementing vector pTCVΩ*abx1*, the overexpressing vectors pTCVΩP_cyl+__*abx1* or pTCVΩP_tet__*abx1*, the negative control vectors pTCVΩP_cyl+__*gbs1037* or pTCVΩP_cyl+__*EGFP* were spotted onto TH, Granada, and blood agar plates containing erythromycin. An overexpression mutant where the *abx1* chromosomal promoter was replaced by the strong P_cyl+_ promoter (ΔP_abx1_::P_cyl+_) was similarly spotted on agar plates without antibiotic. TH and Granada plates were photographed after 24–36 h of growth, while blood agar plates were photographed after 16 h of growth at 37°C.

Screening of a collection of random *Himar1* insertional mutants derived from the NEM316 wild-type (WT) strain allowed us to identify mutants that mapped in the *cyl* operon [40]. We extended this approach with approx. 2,500 new *Himar1* mutants screened for hemolytic activity on blood agar plates and for pigmentation on Granada agar, a specific medium that stabilizes the GBS pigment [42]. We focused our analysis on four mutants displaying a strong decrease in pigmentation and hemolytic activity on Granada and Blood agar plates (see Figure 1A for two of these four phenotypically identical mutants). The four mutants have unique and independent *Himar1* insertions in the uncharacterized gene *gbs1532* as revealed by direct sequencing on chromosomal DNAs. This 921-bp gene encodes a 306 amino acid protein annotated as a hypothetical protein or putative protease (NCBI ref. seq. NP_735969). Transposon insertions of the four *Himar1* mutants were in positions 85, 347, 625, and 632 in the DNA coding sequence, leading to truncated proteins at codons 29, 116, 209, and 211, respectively.

Blast similarity searches, domain analyses, and topology prediction (Figure S1) revealed that Gbs1532 belongs to a conserved superfamily of putative membrane-bound metalloproteases related to eukaryotic CaaX proteases [35], [36]. The characteristic domain of this superfamily of more than 5,500 proteins is the Abi-domain (Pfam02517). To our knowledge, none of the bacterial Abi-domain proteins are functionally characterized to date [36]. We therefore named the translated *gbs1532* gene product Abx1, for Abi-domain protein related to CaaX protease, and renamed the corresponding gene accordingly (*i.e.*, *abx1*). This *abx1* gene belongs to the core genome of all sequenced GBS strains [43], [44] and 6 other genes coding for Abi-domain proteins similar to Abx1 are present in their genomes (Figure S1).

To confirm that *abx1* is required for pigmentation and hemolysis, we constructed in-frame deletion mutant (Δ*abx*1) in the NEM316 WT strain. The Δ*abx1* mutant displayed the same phenotypes as the *Himar1* mutants (Figure 1B). Furthermore, pigment production and hemolysis were restored upon ectopic expression of *abx1* transcribed from its own promoter (Figure 1A and 1B: pTCVΩ*abx1* complementing vector) confirming that *abx1* was responsible for the observed phenotypes.

### Overexpression of Abx1 increases hemolysis and pigment production

Wild-type or Δ*abx1* mutant strains expressing Abx1 from the complementing plasmid pTCVΩ*abx1* were more pigmented and more hemolytic than the control strains (Figure 1B). This suggests that the expression level of Abx1 might be limiting for pigment production and hemolysis. To test this hypothesis, we cloned *abx1* downstream of two constitutive promoters with different strength (P_tet_ and P_cyl+_) to generate the overexpressing vectors pTCVΩP_tet__*abx1*, and pTCVΩP_cyl+__*abx1*, respectively. Furthermore, we engineered the WT strain to replace the chromosomal P_abx1_ promoter by the P_cyl+_ promoter (ΔP_abx1_::P_cyl+_). All recombinant strains carrying the different overexpression forms of *abx1* displayed hyper-hemolysis on blood agar and hyper-pigmentation on Granada agar (Figure 1C). Determination of the hemolytic titer of the different mutants and quantification of the corresponding *abx1* transcription levels further confirmed the link between hemolysis and *abx1* expression (Table 1). Remarkably, the pigmentation level also correlated with the strength of the *abx1* upstream promoter (P_tet_>P_cyl+_>P_abx1_) on TH agar, a medium where pigmentation is usually not observed (Figure 1C). Overexpression of Gbs1037, the closest Abx1 homolog in GBS (40% identity over 222 residues, E-value = e-11; Figure S1) and of the unrelated EFGP (Enhanced Green Fluorescence Protein) did not modify pigmentation and hemolysis of NEM316, underlining the specific activity of Abx1 (Figure 1C). The activity of Abx1 is a conserved feature within the GBS species since *abx1* deletion or overexpression in several clinical strains (serotypes Ia, 515; Ib, H36B; II, 18RS21; III, BM110; and V, 2603V/R; [44]) has a strong effect on their hemolytic activities and pigmentation (Figure S2).

**Table 1:** Hemolytic titer is dependent of *abx1* expression level.

Strain	Hemolytic titer	*abx1* expression*
WT+pTCV	1	1.0 (+/−0.1)
WT+pTCVΩ*abx1*	4–8	2.4 (+/−0.2)
WT+pTCVΩP_cyl+__*abx1*	16–32	13.2 (+/−1.3)
WT+pTCVΩP_tet__*abx1*	32	24.3 (+/−4.9)
WT+pTCVΩP_cyl+__*gbs1037*	0.5–1	n.d.
WT	1	1.0 (+/−0.1)
ΔP_abx1_::P_cyl+_	16–32	4.0 (+/−0.5)

* As measured by qRT-PCR and reported as expression ratio against the WT strain. Mean +/− SD of three independent experiments. n.d. = not determined.

### Induction of hemolysis by Abx1 is not achieved by prenylation- or protease-activity

**Figure 2:**
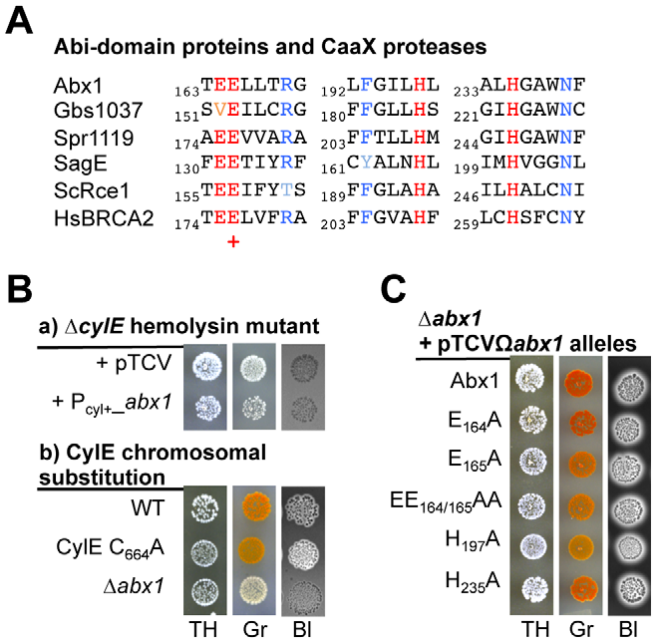
Abx1 function is dependent of the CylE ß-hemolysin through a protease-independent activity. (A) Abx1 is a member of a large family of proteins related to eukaryotic CaaX proteases [35], [36]. The three most conserved motifs of the Abi-domain (Pfam02517) are depicted for Abx1 and Gbs1037 from *S. agalactiae*, Spr1119 from *S. pneumoniae*, SagE from *S. pyogenes* and for two eukaryotic CaaX proteases, the budding yeast ScRCE1 and the human HsBRCA2. Conserved amino acids essential for CaaX protease activity are in red with the putative catalytic glutamic acid (E) residue shown by a plus (+). Figure S1 provides additional information on the Abi-domain family. (B) The CylE hemolysin is essential for Abx1 function on hemolysis and pigmentation. GBS hemolysis and pigmentation were observed on TH, Granada (Gr) and Blood agar (Bl). Strains are: (a) in-frame *cylE* deletion mutant (Δ*cylE*) with the empty vector pTCV or the *abx1* overexpression vector pTCVΩP_cyl+__*abx1*; and (b) WT, *abx1* deletion mutant (Δ*abx1*) and a mutant with a cysteyl to alanyl substitution in the CaaX motif of CylE (CylE C_664_A), *i.e.*, the putative prenylation site of CylE. (C) Conserved amino acids critical for CaaX protease activity are not required for Abx1 activity. The non-hemolytic and apigmented Δ*abx1* mutant were complemented with pTCVΩ*abx1* vectors encoding a wild-type allele (Abx1) or several alanine substitution alleles in the predicted critical glutamic acid (E) or histidine (H) residues.

In eukaryotes, Abi-domain-containing proteins (Pfam02517) are CaaX prenyl proteases involved in protein prenylation [35], [36], [45], [46]. These transmembrane proteases remove the carboxy-terminal aaX tripeptide of a protein after the addition of an isoprenyl group on the cysteyl residue of the CaaX motif. One of the main features of the Abi-domain is the presence of four predicted core transmembrane helical segments (labeled TMH1–4) containing conserved active-site residues (Figure S1D). The overall topology and the critical residues for protease activity are conserved in Abx1 (Figure 2A and S1). To date, prenylation was never described in prokaryotic cells but, intriguingly, the CylE hemolysin contains a cysteyl residue at the fourth position from its carboxylic end (*i.e.*, the critical residue of the CaaX motif [46]).

We tested whether CylE could be post-translationally modified by a prenylation-like mechanism involving Abx1. Overexpression of Abx1 using pTCVΩP_cyl+__*abx1* in the non-hemolytic and non-pigmented Δ*cylE* mutant did not increase hemolysis or pigmentation (Figure 2B), confirming that Abx1 function is strictly dependent on the presence of a functional CylE. We then substituted the cysteyl residue of CylE located at the fourth position from its carboxy-terminal end by an alanyl (CylE C_664_A). This mutation did not alter pigmentation and hemolysis significantly (Figure 2B), which suggests that CylE is not prenylated on this cysteyl, and consequently that Abx1 might not be a CaaX protease. To further test whether Abx1 has protease activity, we replaced the conserved residues (glutamyl at positions 164 and 165 and histidyl at positions 197 and 235) described as critical for this enzymatic activity by alanyl residues (Figure 2A; [47]). All alleles obtained for Abx1 (E_164_A, E_165_A, EE_164/165_AA, H_197_A, H_235_A) restored hemolysis and pigment production when expressed in a Δ*abx1* mutant strain (Figure 2C). Hence, the putative protease activity of Abx1 is not necessary for the CylE dependent phenotypes.

### Abx1 and CovR, the major regulator of virulence gene expression, are functionally linked

**Figure 3:**
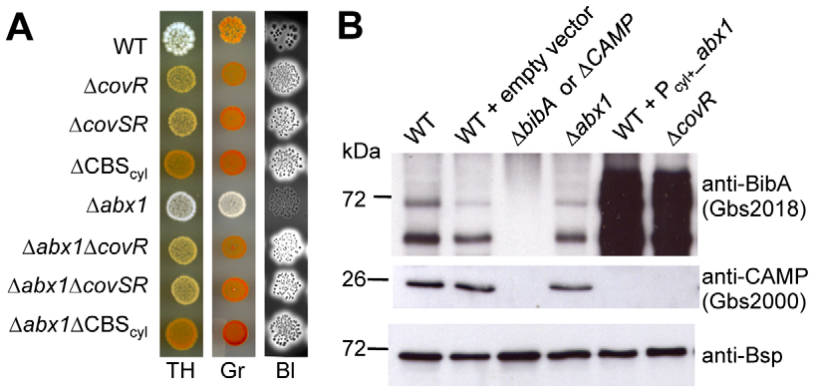
The two-component system CovSR is epistatic to Abx1. (A) Control of hemolysin and pigment production by Abx1 is dependent on the two-component system CovSR. Phenotypic comparison of WT and *abx1* mutant without or with mutations in CovR (Δ*covR*), CovSR (Δ*covSR*), and in the CovR binding sites of the *cyl* operon (ΔCBS_cyl_), as assayed on TH, Granada (Gr) and Blood agar (Bl) plates. (B) CovR-regulated genes are similarly expressed in *abx1*-overexpressing and *covR*-deletion mutants. Immunoblots of the surface-exposed adhesin BibA (Gbs2018) and secreted CAMP factor (Gbs2000) in cell wall extracts and concentrated supernatants, respectively. The Bsp protein was used as a loading control. Specificities of BibA and CAMP factor antibodies were confirmed with extracts from deletion mutants of the corresponding genes (negative controls).

Abx1 activity is dependent of CylE but appears to be independent of a CylE post-translational modification. To decipher the relationship between Abx1 and *cyl*-dependent hemolysis and pigment production, we took advantage of the previously described ΔCBS*_Cyl_* mutant where the CovR binding sites (CBS) in the promoter of the *cyl* operon were deleted [40]. Since CovR acts as a transcriptional repressor of the *cyl* operon, deletion of CBS*_Cyl_* leads to strong constitutive expression of the *cyl* operon. Consequently, the ΔCBS*_Cyl_* mutant is hyperhemolytic and hyperpigmented in all tested conditions (Figure 3A). Whereas *abx1* deletion in a WT background abolishes pigmentation and hemolysis, deletion of *abx1* in a ΔCBS*_Cyl_* background (Δ*abx1*ΔCBS*_Cyl_*) did not affect the hyper-hemolytic and hyper-pigmented phenotypes of the parental ΔCBS*_Cyl_* strain (Figure 3A). This result suggests that Abx1 acts upstream of CovR-mediated transcriptional inhibition of the *cyl* operon.

To confirm this epistatic interaction, we inactivated *covR* (*gbs1672*) and the complete *covSR* two-component system (*gbs1671–1672*) in the WT and Δ*abx1* backgrounds. As expected, inactivation of *covR* or *covSR* in the WT strain led to hyper-pigmentation and hyper-hemolysis (Figure 3A). Similarly, inactivation of CovR or CovSR in the Δ*abx1* background restored pigmentation and hemolysis to levels similar to those observed in the Δ*covR* and Δ*covSR* mutants (Figure 3A). This suggests that Abx1 is an inhibitor of CovR activity. Consistently, Western blot analysis of the adhesin BibA and CAMP factor whose expression are negatively and positively regulated by CovR [11], [13], respectively, revealed similar expression patterns in the Δ*covR* and *abx1* overexpression mutants: inactivation of CovR or overexpression of *abx1* dramatically increased BibA production and abolished secretion of the CAMP factor (Figure 3B). These results demonstrate that Abx1 activity is not restricted to pigmentation and hemolysin production, and suggest an antagonist function on CovR activity.

### Abx1 inhibits CovSR signaling by antagonizing CovS

**Figure 4:**
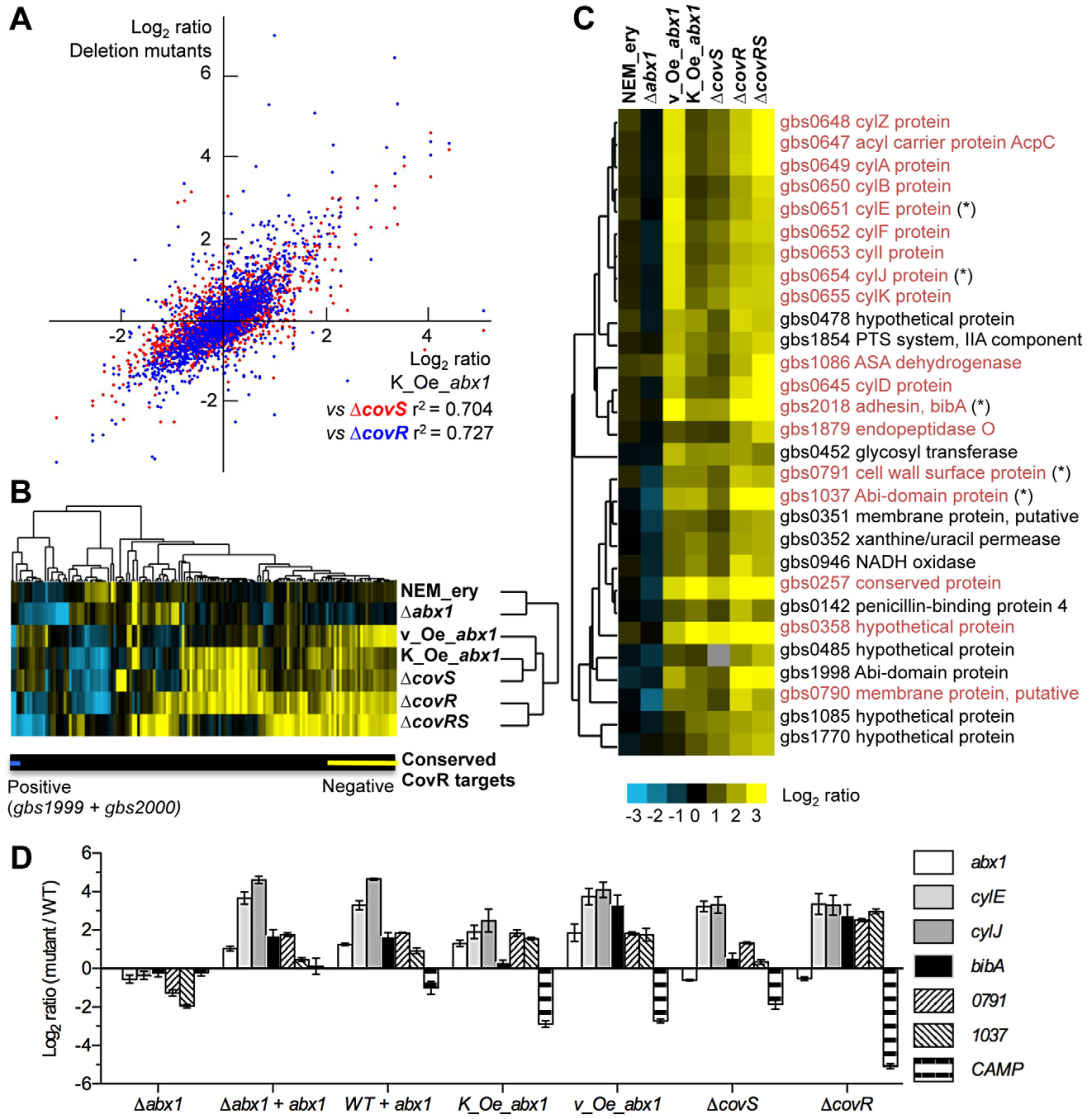
Abx1 inhibits the CovSR system. Transcriptome analysis of deletion (Δ*abx1*, Δ*covS*, Δ*covR*, and Δ*covRS*) and overexpressing (Oe*_abx1*) mutants. The latter mutants were obtained either by chromosomal replacement of the *abx1* WT promoter by the strong constitutive promoter P_cyl+_ (K_Oe_*abx1*) or by using the overexpression vector pTCVΩP_cyl+__*abx1* (v_Oe*_abx1*). The WT strain with the empty vector (NEM_ery) was included to take into account the potential effect of erythromycin (10 µg/ml) used to maintain the *abx1* overexpression vector. (A) Pairwise comparisons of Log_2_ expression ratios for 1,905 genes between *abx1* overexpressing mutant (K_Oe_*abx1*) and Δ*covR* (blue dots) or Δ*covS* (red dots) deletion mutants. Pearson's correlations (r^2^) were calculated to estimate similarities in gene expression change. (B) Heatmap of the genes (N = 147) with an absolute log_2_ ratio >2 in at least one strain. Gene expression changes were color-coded (blue = down; yellow = up). Hierarchical clustering (uncentered; average linkage) was done for genes (upper tree) and strains (right tree). Genes of the CovSR regulon conserved in 3 sequenced GBS strains (NEM316, A909, and 2603V/R) are highlighted below the heatmap (blue line = down-conserved; yellow line = up-conserved). (C) Highlight of the gene cluster in (B) containing the conserved negatively regulated genes of the CovSR regulon (in dark red letters). The gene tree is shown on the left of the heatmap and the corresponding gene identifiers and protein annotations are indicated on the right. Genes marked with a star were selected for confirmation of gene expression by qRT-PCR. Enlarge version of (B) and (C) heatmaps at a lower threshold ( = absolute log_2_ ratio >1 in at least one strain) are presented in supplementary figure S3. (D) qRT-PCR expression analysis of selected genes in *abx* and *cov* mutants. The relative expression level of *abx1*, of 5 negatively-regulated CovR genes (*cylE*, *cylJ*, *bibA*, *gbs0791 and gbs1037*), and of one positively-regulated CovR gene (*cfb* coding the CAMP factor) were measured in Δ*abx1*, Δ*covS*, and Δ*covR* deletion mutants, in the WT and Δ*abx1* mutant containing the *abx1* complementing vector (+ *abx1*; plasmid pTCVΩ*abx1*), and in two *abx1* overexpressing mutants (K_Oe*_abx1* = chromosomal *abx1* overexpression; v_Oe*_abx1* = vector-based *abx1* overexpression with the pTCVΩP_cyl+__*abx1* plasmid). Results are the relative expression level between mutant and WT strains expressed as the means value (+/− SEM) from three independent cultures in triplicates.

To decipher the relationship between Abx1 and the CovSR two-component system, we compared the transcriptomes of Δ*covS*, Δ*covR* and Δ*covSR* mutants with the Δ*abx1* deletion mutant and two *abx1* overexpression mutants (v_Oe_*abx1* = vector based *abx1* overexpression with the pTCVΩP_cyl+__*abx1* plasmid; and K_Oe_*abx1* = chromosomal *abx1* overexpressing strain ΔP_abx1_::P_cyl+_). Pairwise comparisons of Log_2_ expression ratios (Table S1, N = 1,905 genes) revealed highly similar expression changes between the strain carrying the chromosomal overexpression of *abx1* (K_Oe_*abx1*) and the Δ*covS* and Δ*covR* deletion mutants (Figure 4A: Pearson correlation = 0.704 and 0.727, respectively). Strikingly, the transcriptome profiles of the *abx1* overexpression and Δ*covS* deletion mutants form a distinct cluster as revealed by hierarchical clustering analysis (Figure 4B and S3A).

Detailed analyses revealed a cluster of genes containing the direct and conserved targets that are negatively regulated by CovR (Figure 4C and S3B: N = 29 and 40 genes with expression change log_2_>2 and >1 in at least one strain, respectively). Expression of nearly all of these genes, including the *cyl* operon (*gbs0644*–*gbs0655*) and the *bibA* gene (*gbs2018*), were dependent on the expression level of *abx1*. Moderate overexpression of *abx1* in the chromosome (ΔP_abx1_::P_cyl+_; 3.7 fold change by microarrays) induced a transcriptome response highly similar to that of the Δ*covS* mutant (Figure 4C). Greater overexpression of *abx1* (pTCVΩP_cyl+__*abx1*; 9.1 fold change by microarrays) further increased expression of the direct-CovR regulated gene set to a level close to that observed in a Δ*covR* mutant (Figure 4C). In contrast, downregulation below the significant threshold is observed in the Δ*abx1* mutant (Figure 4C). qRT-PCR analysis on a set of 6 genes regulated by the CovSR system confirmed gene expression changes identified by microarray analysis (Figure 4D). Moreover, *bibA* and *CAMP* factor transcription levels in the Δ*covR* deletion and *abx1* overexpression mutants are in accordance with the corresponding protein expression levels as observed by Western analysis (Figure 3B). Taken together, the similarities between *abx1* overexpression mutants and *cov* deletion mutants suggest that Abx1 exerts a specific effect on the CovSR two-component system, most likely by acting as a CovS antagonist.

### Abx1 interacts directly with the histidine kinase CovS

**Figure 5:**
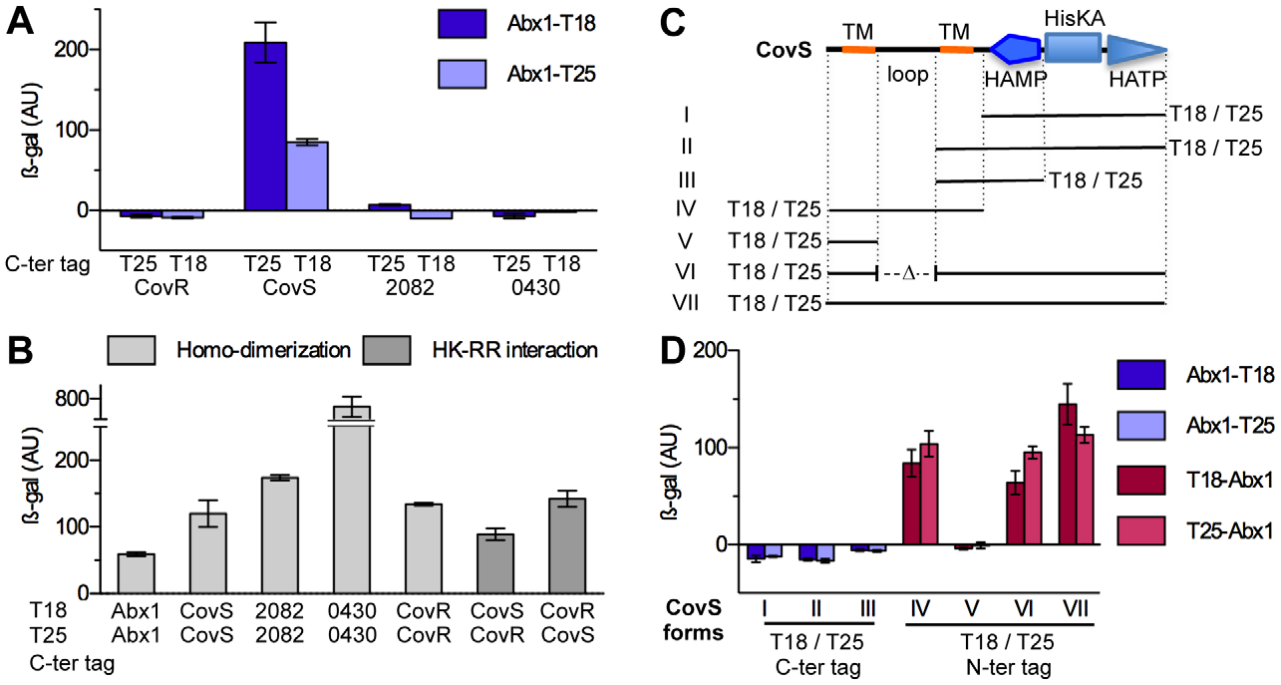
Abx1 physically interacts with the HK CovS. The physical interactions between proteins were assayed using a two-hybrid system in *E. coli*. Fusion proteins were constructed with the T18 or the T25 adenylate cyclase fragments. The activity of the ß-galactosidase cAMP-dependent reporter gene, expressed in arbitrary units (AU), was determined as described in Materials and Methods. Means and standard deviations are calculated from at least three independent cultures. (A) Interactions between Abx1 and the transcriptional regulator CovR, the histidine kinase CovS, and two others GBS histidine kinase (Gbs2082 and Gbs0430). The T18 and the T25 tags are located at the C-terminal end of tested proteins. Interactions of Abx1-T18 (dark blue) were measured against the indicated T25 tag protein. Reciprocally, interactions of Abx1-T25 (light blue) were measured against the indicated T18 tag protein. (B) Homo-dimerizations were measured by the interaction between the T18 and T25 fusions of the same proteins and are show with light grey bars. The specific interaction between the HK CovS and its RR CovR is shown with dark grey bars. (C) Schematic representation of the CovS histidine kinase domains and of the truncated alleles used in (D). The amino-terminal part of CovS (residues 1–211) is made of two transmembrane (TM) domains flanking an extracellular loop. The cytoplasmic carboxy-terminal part of CovS (residues 211–501) contains the typical HAMP, HisKA and HATP regulatory and catalytic domains. Truncated alleles (I to VI) were tag with either T18 or T25 at the C-terminal end (I to III) or at the N-terminal end (IV to VI). A full length CovS allele tagged at the N-terminal end (VII) was added as a control. (D) Interactions between Abx1 and truncated alleles of CovS. The CovS alleles with a C-terminal tag were tested against the corresponding Abx1 tagged at its C-terminal end (blue). Similarly, the CovS alleles with a N-terminal tag were tested against the corresponding Abx1 tagged at its N-terminal end (red).

Our results suggest an inhibitory function of Abx1 on CovS activity or that Abx1 protects or sequesters CovR, impeding its phosphorylation by CovS either directly or indirectly. To discriminate between these possibilities, we used a bacterial two-hybrid system [48] to test the physical interactions of Abx1 with different putative partners. This showed that Abx1 is able to form homodimers and that it interacts indeed with CovS but not with CovR (Figure 5A and 5B). The Abx1-CovS interaction is specific, as Abx1 did not interact with two other GBS HKs that are similar to CovS (Figure 5A: Gbs2082 and Gbs0430). To define the regions involved in the Abx1-CovS interaction, we tested the interaction of Abx1 with different domains of CovS (Figure 5C). These experiments showed that Abx1 interacts specifically with the amino-terminal part of CovS containing its extracytoplasmic and transmembrane domains (Figure 5D). Deletion of the extracytoplasmic loop of CovS did not impede interaction with Abx1, suggesting that the loop is not directly required for Abx1-CovS interaction (Figure 5D: CovS form VI). No interaction was detected either with the carboxy-terminal part of CovS containing the catalytic domains or with CovS truncated forms containing only one transmembrane domain (Figure 5D). These results suggest that Abx1 interacts with the two CovS transmembrane domains involved in signal processing.

### Abx1 function is dependent on CovS histidine kinase/phosphatase activities

**Figure 6:**
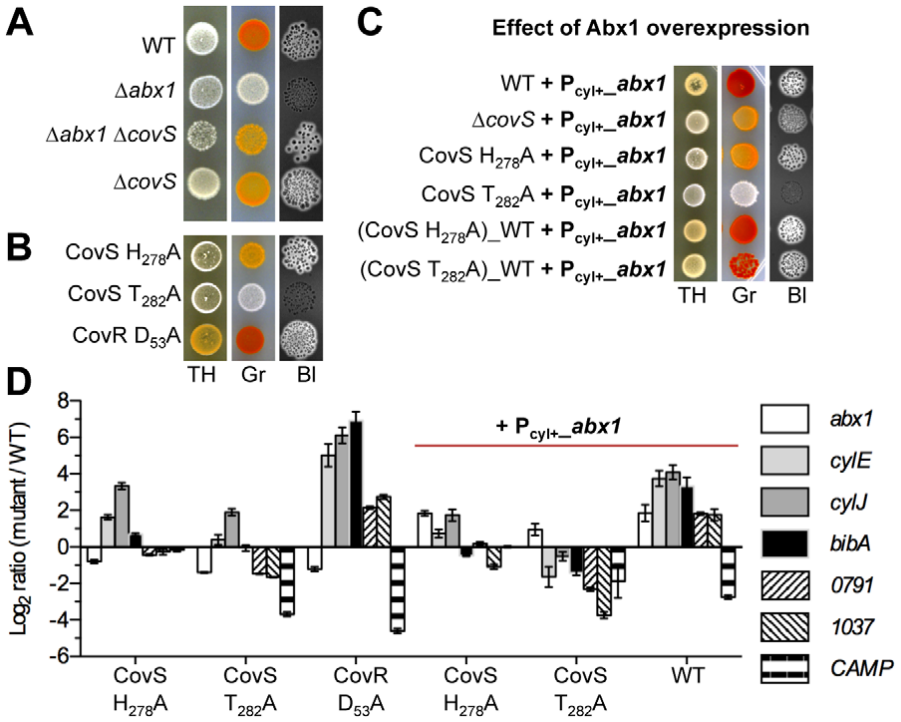
CovS activities are Abx1-dependent. (A) CovS is epistatic to Abx1. Phenotypes of the WT strain, Δ*abx1*, Δ*abx1*Δ*covS*, and Δ*covS* deletion mutants. (B) CovS kinase/phosphatase activities are essential for CovR regulation. Phenotypes of Δ*covS* deletion mutant were compared with those of critical point mutants of CovS (H_278_A and T_282_A) or CovR (D_53_A). The histidine residue number 278 (H_278_) is the conserved phospho-accepting residue essential for the autophosphorylation of CovS. The threonine number 282 (T_282_) is a conserved residue necessary for the phosphatase activity of several HKs. The aspartate number 53 (D_53_) is the conserved residue of CovR phosphorylated by CovS. (C) Abx1 is dependent of CovS activities. Phenotypes of WT, Δ*covS*, and CovS H_278_A or CovS T_282_A alanine substitution mutants overexpressing *abx1* (+P_cyl+_ *_abx1* = plasmid pTCVΩP_cyl+_ *_abx1*). The hyper-pigmentation and hyper-hemolysis associated to *abx1* over-expression in WT strain are suppressed in Δ*covS* and CovS H_278_A mutants. Reversion of CovS H_278_A and T_282_A substitution back to the WT CovS sequence (indicated by _WT) restores the *abx1* over-expression associated-phenotypes. All assays were performed on TH, Granada (Gr) and blood agar (BI) plates. (D) *abx1* overexpression effects are dependent of CovS enzymatic activities. Relative expression levels of selected genes were measured by qRT-PCR in logarithmic growing cells in TH media. Relative expression levels of *abx1*, of five negatively regulated CovR genes (*cylE*, *cylJ*, *bibA*, *gbs0791 and gbs1037*), and of one positively regulated CovR gene (*cfb* coding the CAMP factor) were measured in CovS and CovR alanyl substitution mutant, and in WT and CovS alanyl substitution mutants overexpressing *abx1* (+P_cyl+_ *_abx1* = plasmid pTCVΩP_cyl+_ *_abx1*). Results are means +/− sem from three independent cultures in triplicates.

In agreement with the proposed inhibitory function of Abx1 on CovS, deletion of *covS* in a Δ*abx1* background (Δ*abx1*Δ*covS*) restores pigment production and hemolysis of the non-pigmented and non-hemolytic Δ*abx1* mutant to levels similar to those of the Δ*covS* mutant (Figure 6A). However, as seen previously by transcriptome analysis, the Δ*covS* mutant is not identical to the Δ*covR* mutant. In particular, the Δ*covR* mutant is hyper-pigmented and hyper-hemolytic (see Figure 3A) while the Δ*covS* mutant is only slightly affected in pigmentation and hemolysis compared to the WT strain (Figure 6A).

To explain these observations, we must consider the three common activities of histidine kinases (HK): auto-phosphorylation of the kinase on a histidyl residue, phospho-transfer on an aspartyl residue of the cognate regulator, and phosphatase activity of the HK on its cognate regulator [14]. Deletion of *covS* abolishes the three activities. In this case, cross-phosphorylation of the regulator by small metabolites or by a non-cognate HK can be observed due to the absence of the phosphatase activity of the cognate HK [49], [50]. Thus, in the Δ*covS* mutant, cross-phosphorylation of CovR might enable its partial activation. To gain experimental evidence, we constructed CovS and CovR alanyl substitution mutants in conserved residues critical for each of the three activities associated with HK [14]. First, we targeted the phospho-acceptor histidyl residue H_278_ to obtain an auto-phosphorylation deficient allele of CovS. Second, we replaced the aspartyl residue D_53_ of CovR normally phosphorylated by CovS to abolish phospho-transfer between CovS and CovR [25], [26]. Third, we replaced the CovS threonyl residue T_282_ that was proposed to be specifically involved in HK phosphatase activity [50], [51]. We then used pigmentation and hemolysis production as reporters of CovSR activity (Figure 6B).

The CovR D_53_A mutant displayed a hyper-hemolytic and hyper-pigmented phenotype like the Δ*covR* mutant (Figure 6B), as also observed previously in another GBS strain [25], supporting the model of CovR as a transcriptional repressor under its D_53_^P^ phosphorylated form. Analyses of the CovS H_278_A mutant strain showed phenotypes similar to the Δ*covS* mutant (Figure 6B), suggesting that the H_278_ residue is essential for all three activities, as assumed by a mechanistic model of HK activity [50], [51]. We identified in CovS the conserved threonyl residues specifically required for the phosphatase reaction of several HK [50], [51]. Interestingly, the CovS T_282_A mutant is non-hemolytic and non-pigmented (Figure 6B) indicating that CovR is locked in an active form that fully inhibits the expression of the *cyl* operon. Overexpression of *abx1* in the Δ*covS*, CovS H_278_A or CovS T_282_A mutants did not modify their phenotypes, indicating that Abx1 activity is dependent on CovS kinase/phosphatase activities (Figure 6C). The fact that overexpression of *abx1* in the WT context led to hyper-pigmented and hyper-hemolytic phenotypes, as seen for the Δ*covR* and CovR D_53_A mutants (Figure 6), suggests that Abx1 actively stimulates CovS-dependent dephosphorylation of CovR. Expression level measurements of selected CovR-regulated genes (*cylE*, *cylJ*, *bibA*, *gbs0791*, *gbs1037*, and *cfb* coding the CAMP factor) in CovS and CovR alanyl substitution mutants confirm that the broad effect of *abx1* overexpression depends upon a functional CovS histidine kinase (Figure 6D). Overall, our results support a model where Abx1 inhibits the kinase-competent form and/or stabilizes the phosphatase-competent form of CovS.

### Abx1-CovS and Stk1 converge to regulate CovR activity

**Figure 7:**
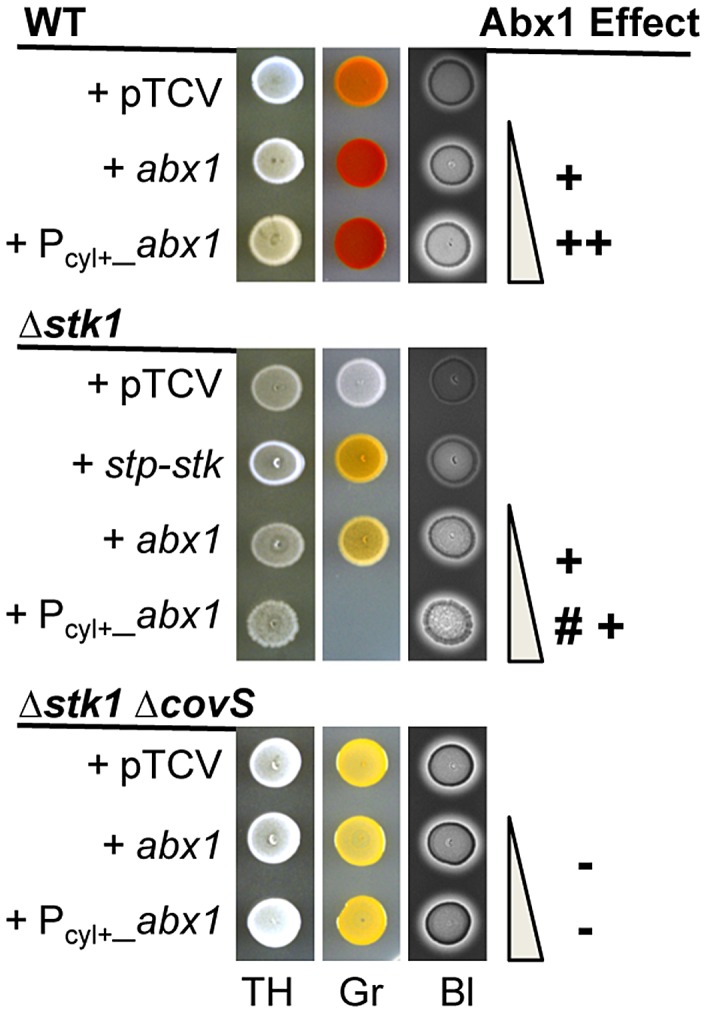
CovR-dependent phenotypes are regulated by convergent signaling pathways mediated by Abx1-CovS and Stk1. Phenotypes associated to the inactivation of the serine/threonine kinase Stk1 in a WT background (Δ*stk1*) and in combination with CovS inactivation (Δ*stk1*Δ*covS*). All strains were transformed with the empty vector pTCV or derivatives. To complement the Δ*stk1* mutant, the two-gene operon containing *stk1* was cloned with its promoter into the pTCV vector (pTCVΩ*stp-stk*). To compare the effect of Abx1 on CovR-dependent phenotypes, the Abx1 complementing vector (pTCVΩ*abx1*) or the Abx1 over-expression vector (pTCVΩP_cyl+_ *_abx1*) were introduced into relevant strains. Increased pigmentation and hemolysis due to the presence of the *abx1* vectors are indicated with a plus sign (+), absence of effect of the *abx1* vectors are indicated by a minus sign (−), and condition-dependent toxicity of *abx1* overexpression specifically on Granada medium is indicated by a mark (#). All assays were performed on TH, Granada (Gr) and blood agar (BI) plates.

An additional regulator of the CovSR system is the eukaryotic-like serine/threonine kinase Stk1. It has been shown *in vitro* that Stk1 regulates CovR negatively by direct phosphorylation of the threonyl residue at position 65 (T_65_) [25], [34]. The CovR T_65_ phosphorylation by Stk1 has been proposed to be mutually exclusive of CovR D_53_ phosphorylation by CovS [25]. Consistently, the Δ*stk1* (Δ*gbs0307*) mutant is strongly and negatively affected in pigment production and hemolytic activity (Figure 7), in agreement with an inhibitory role of Stk1 on CovR activity.

To learn whether Abx1 and Stk1 function together, we tested if Abx1 and Stk1 interact using the bacterial two-hybrid system. No interaction was observed (data not shown). Furthermore, pigmentation and hemolysis were restored in the non-pigmented and non-hemolytic Δ*stk1* strain upon increasing *abx1* copy number with plasmid pTCVΩ*abx1* (Figure 7). This indicates that Abx1 is able to bypass CovR activation observed in the absence of Stk1. However, pigmentation and hemolysis levels of the Δ*stk1* strain carrying the pTCVΩ*abx1* vector are below those of the WT strain (Figure 7). This result suggests that *abx1* overexpression needs a functional Stk1 to fully inhibit CovR activity.

**Figure 8:**
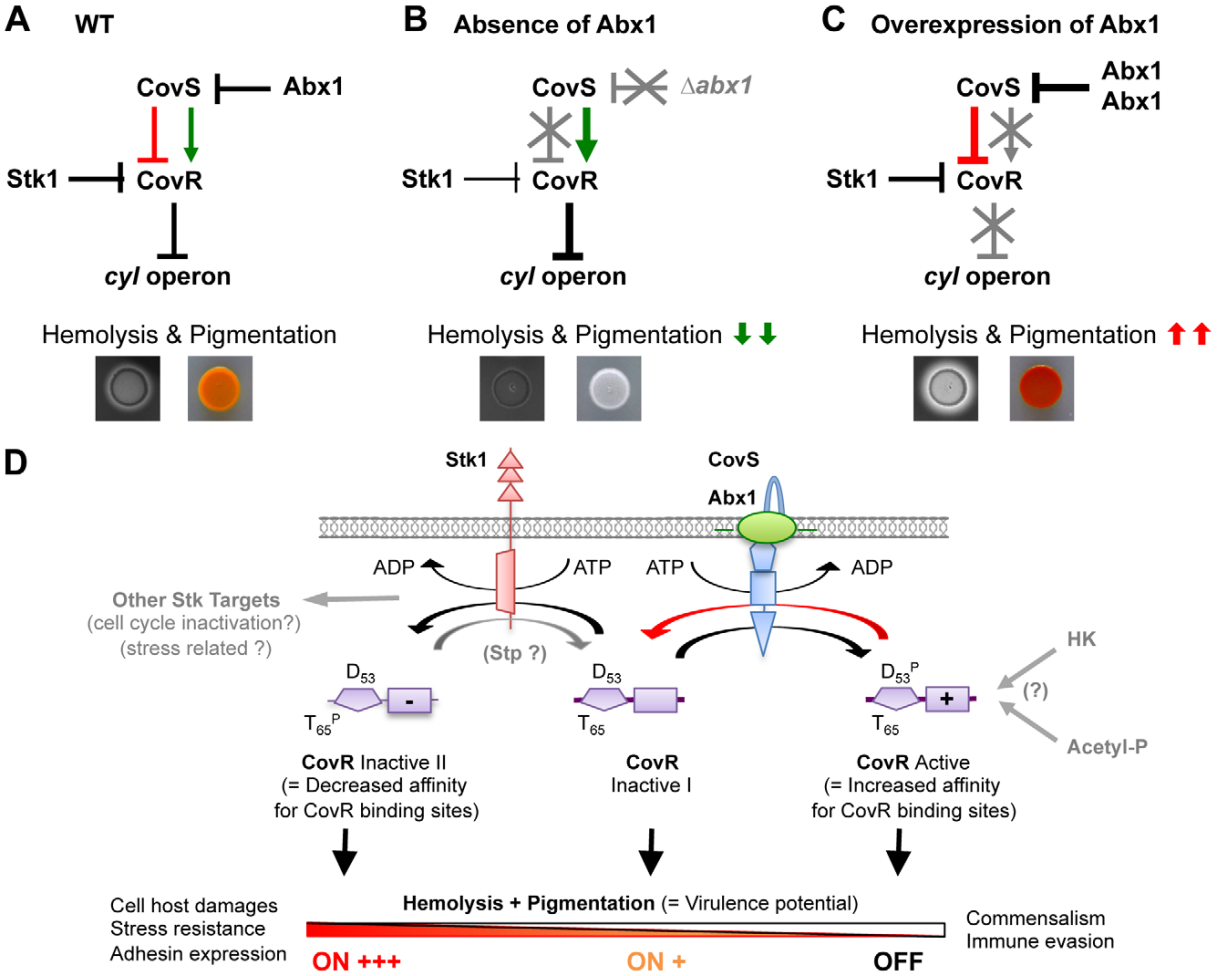
Proposed model of virulence gene regulation by the CovS/Abx1/Stk1/CovR network. (A) Simplified wiring diagram of the regulatory network controlling GBS hemolysin and pigment production. In a WT context, the Abi-domain protein Abx1 maintains the equilibrium between the kinase (green arrow) and the phosphatase activity (red lines) of the HK CovS. Activation of the RR CovR by CovS-dependent phosphorylation increases its inhibitory activity on *cyl* operon transcription. Inhibition of CovR is dependent on its dephosphorylation by CovS and its phosphorylation by the Stk1 serine/threonine kinase. In this condition, the *cyl* operon is expressed at a level defining the WT β-hemolytic and pigmentation phenotype. (B) In the absence of Abx1, the HK CovS is locked in its kinase-competent form that activates CovR, thus inhibiting the expression of the *cyl* operon. Phosphorylation of CovR by CovS precludes the Stk1-dependent CovR phosphorylation, leading to minimal CovR inhibition by Stk1. In this condition, GBS are non-hemolytic and non-pigmented. (C) In the presence of an excess of Abx1, the kinase-competent form of CovS is inhibited and/or the phosphatase-competent form is stabilized. The kinase Stk1 is necessary to fully inactive CovR, thereby allowing a strong expression of the *cyl* operon. In this condition, GBS are hyper-hemolytic and hyper-pigmented. (D) An integrated model for CovSR signaling with modular schematization of CovS (blue), Abx1 (green), Stk1 (red) and CovR (purple). The CovR activity reflects the equilibrium between the mutually exclusive ATP-dependent phosphorylation of two critical residues: threonyl 65 (T_65_) by Stk1 and aspartyl 53 (D_53_) by CovS, as proposed in [15], [25]. This equilibrium is dependent on Abx1, which is essential for CovS-dependent inactivation of CovR (red arrow). Modulation of CovR affinities for DNA by phosphorylation regulates the expression of target genes, among which those necessary for stress resistance, host adhesion and damages characteristic of invasive bacterial forms. Potential connections on the core of the signaling network are in grey (other Stk1 targets, putative Stp phosphatase activity, putative interaction of CovS-Abx1 with other HKs, putative CovR phosphorylation by small phosphate donor as acetyl-phosphate).

Finally, a condition-dependent toxicity was observed when the overexpressing pTCVΩP_cyl+__*abx1* vector was introduced into the Δ*stk1* mutant. Specifically, strong overexpression of *abx1* in Δ*stk1* is toxic on Granada agar (Figure 7). This phenotype is CovS-dependent as toxicity is alleviated in a Δ*stk1*Δ*covS* double mutant, which is unresponsive to *abx1* overexpression (Figure 7). This result reinforces the link between Abx1 and a functional CovS. In addition, the Δ*stk1*Δ*covS* mutant is locked in an intermediate phenotype (Figure 7), revealing a partial inhibition of CovR in the absence of CovS. Thus, CovS appears necessary for complete activation of CovR in the absence of Stk1, most likely by an active CovR D_53_ phosphorylation. As shown in the model depicted Figure 8, we propose that the regulatory function of Abx1 is absolutely dependent on CovS but also depends on the serine/threonine kinase Stk1 to control CovR activity by a convergent signaling pathway.

### *abx1* expression level is critical for virulence

**Figure 9:**
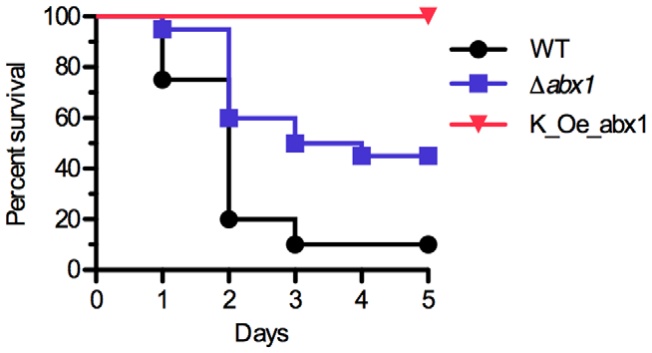
Virulence of *abx1* mutants. Mortality curves in neonate rats infected with the WT strain (black circle), the Δ*abx1* deletion mutant (blue square), and the K_Oe_abx1 overexpressing mutant (red triangle). Two days rat pups were inoculated by intraperitoneal injection of 5×10^6^ bacteria. The results shown are the mean of two independent experiments (2×10 rats per group).

The CovSR two-component system is a master regulator of GBS virulence gene expression. In order to assess the role of Abx1 in GBS virulence, neonatal rat pups were infected by intraperitoneal injection of 5×10^6^ bacteria of either the Δ*abx1* deletion or *abx1* overexpressing mutants. In this model, both mutants exhibited reduced virulence compared to the parental WT strain (Figure 9). It is noteworthy that the *abx1* overexpression mutant is totally avirulent (100% survival) and that neonates did not display any sign of illness during the course of two independent experiments (data not shown). These results show that *abx1* expression at an appropriate level is required to develop GBS infection.

## Discussion

The CovSR two-component system (TCS) is the major regulator of virulence gene expression in GBS. In this study, we identified the Abx1 transmembrane protein as essential and limiting for CovSR activity due to its interaction with the CovS histidine kinase (HK). Regulation of HK activity by an interacting protein is increasingly recognized when studying TCS signaling in a cellular context [31]–[33]. Instead of being a linear signaling pathway, our data show that the CovSR TCS is embedded in a regulatory network involving several regulatory proteins, including the Abi-domain protein Abx1.

### Abx1 as a histidine kinase regulator

We first observed that Abx1 is required for hemolysin and pigment production in GBS and that *abx1* overexpression in a WT background increases hemolysin and pigment production in a dose-dependent manner. These *abx1* effects depend upon a functional CovSR pathway, which directly controls expression of the *cyl* operon. In agreement with a regulatory activity of Abx1 on the CovSR system, modulation of *abx1* expression affected not only hemolysin/pigment production but also production of the adhesin BibA and the CAMP factor, two direct targets of CovR [11], [13]. Indeed, genome-wide transcriptome profiling suggested a specific and antagonistic activity of Abx1 on the CovSR system.

By combining genetic analysis and protein-protein interactions, we show that Abx1 acts through the HK CovS to control the activity of CovR. The transmembrane protein CovS is the cognate HK of CovR. However, in contrast to the majority of TCS, CovR is mainly a transcriptional inhibitor when phosphorylated on the conserved D_53_ residue and CovS is assumed to be primarily a phosphatase necessary to relieve repression of virulence genes during infection [11]–[13], [24]. Our data support this regulatory logic (*i.e.*, the inactivation by CovS of the CovR inhibitor) but show in addition that Abx1 exerts a cellular control on the dual kinase/phosphatase activity of CovS (Figure 8). By using the *cyl* operon as reporter of CovR activity, we observed constitutive CovR activation with a CovS allele (CovS T_282_A) mutated in the conserved threonyl residue specifically involved in the phosphatase activity of several other HKs [50], [51] or by deletion of Abx1 (Figure 8B). Conversely, CovR can be inactivated by a D_53_A substitution, mimicking a non-phosphorylated allele, or by overexpression of Abx1 (Figure 8C). Furthermore, all of these Abx1 effects are dependent on a functional CovS. Taken together, these results suggest that the level of Abx1 is critical to control the equilibrium between the kinase and phosphatase activities of CovS.

It is noteworthy that inactivation of both CovS kinase and phosphatase activities, either with a CovS H_278_A allele or by deleting *covS*, led to intermediate phenotypes compared to those obtained by CovR inactivation. This pattern has been observed previously in GBS [13] and GAS for the CovSR ortholog [24], [52], [53]. In this condition, it is likely that CovR is partially activated by cross-phosphorylation, either by small metabolites like acetyl-phosphate or by a non-cognate HK. The specific phosphatase activity of CovS avoids inappropriate activation of CovR by other pathways, a general mechanism to insulate TCS signaling pathways [49].

Importantly, HK phosphatase activity is not simply the reverse reaction of the kinase activity [50], [51]. The two activities involve different, but potentially overlapping, catalytic residues [50] and are mutually exclusive since they depend on different conformations of HK homodimers [14]. Both Abx1 and CovS are transmembrane proteins. We found that Abx1 interacts directly with CovS input domains (*i.e.*, the extracellular and transmembrane domain), and ompacts CovS output domains (*i.e.*, the catalytic cytoplasmic domain). Current models of HK activity regulation are based on different conformational states of HK homodimers [14]. It is therefore most likely that Abx1 interferes with the dynamic process of CovS conformational changes propagated along the whole proteins to control the kinase/phosphatase switch [14]. In the absence of Abx1, CovS appears to be locked into a kinase-competent conformation while overexpression of Abx1 will result in stabilization of the phosphatase-competent conformation (or destabilization of the kinase-competent form). Of note, the CovS extracellular loop is not necessary for the physical interaction with Abx1 and it is therefore likely that the main domains of CovS interacting with Abx1 are the transmembrane domains flanking this loop. These HK transmembrane domains control the conformational rotation of the cytoplasmic subunits of HK homodimer by a rotation or a piston mechanism [54], [55]. Thus, intra- and inter-molecular interactions interfering with this dynamic process will impact the catalytic activity by blocking HKs in a kinase or a phosphatase form [56], [57].

Positive and negative regulation of HK activity by physical interactions with transmembrane regulators has been described in both gram-negative and gram-positive bacteria [31], [32]. Members of this category are mainly small hydrophobic peptides, like the PhoQ-B1500/SafA [58], PhoQ-MgrB [59] and EnvZ-MzrA [60] pairs in enteric bacteria, or proteins with a single transmembrane domain, like the YycH and YycI proteins regulating the essential HK YyfG in *Bacillus subtilis* [61], [62]. In particular, structural modeling suggests that the YyfG HK is inactivated by its interactions with YyfH and YyfI in the membrane environment [62]. In analogy, this suggests a mechanism of CovS inactivation by its interaction with the eight predicted transmembrane spanning domains of Abx1 (Figure S1).

### The CovS-Abx1 signaling module

Abx1 controls GBS virulence gene expression by regulating CovS activity, *via* a mechanism independent of the direct sensing of an environmental signal by CovS. This cellular regulation of TCS by a “third” component raises the question of how this additional regulator is itself regulated [32], [33]. The HK regulator may set up a feedback loop when located in the same operon as the HK, as for *B. subtilis* YycHI [61], [62], or it may act as a TCS connector when its transcription is controlled directly by another TCS [31] as for *E. coli* B1500/SafA, MgrB, and MzrA peptides mentioned above [58]–[60]. We did not find evidence that *abx1* transcription is directly controlled by CovR (data not shown) but cannot exclude transcriptional regulation by an as yet unidentified RR.

An alternative hypothesis would be an allosteric regulation and/or a scaffolding function of Abx1 [33]. Regulation of HK activity by signal(s) not directly recognized by the HK but sensed and/or transmitted by its interactor(s) might be a common mechanism, as already described for antimicrobial cationic peptide resistance modules in *Firmicutes* [63], [64] and for HK-HK complex formation in *Pseudomonas aeruginosa* [65]. Until now, it is not known how CovS activity is regulated in GBS. In GAS, the CovS ortholog (displaying 50.3% identity with the GBS CovS) is thought to be a direct sensor of magnesium and of the human antimicrobial peptide LL-37 [22], [29], [30]. However, only two Abi-domain proteins are present in GAS genomes and neither is an Abx1 ortholog (Figure S1). Although the biological function of the CovS orthologs in virulence is conserved [19], [20], GAS and GBS inhabit different ecological niches (nasopharynx and skin for GAS; gut and vagina for GBS), suggesting a species-specific response by CovS. The identification of conditions bypassing the Abx1 effects in GBS will be valuable for characterizing the putative signal(s) directly sensed by CovS.

We found that activity of the Abx1-CovS module depends upon a second signaling pathway mediated by the serine/threonine kinase Stk1 [25], [34]. The two pathways converge on CovR to regulate virulence gene expression (Figure 8). *In vitro*, direct phosphorylation of the CovR T_65_ threonyl residue by Stk1 interferes negatively with phosphorylation of the conserved D_53_ aspartyl residue and decreases CovR affinity for its target promoters [25], [34]. By genetic analysis, we found that the Abx1-CovS and the Stk1 signaling pathways are dependent on each other to fulfill complete activation or inhibition of CovR. CovR phosphorylation on T_65_ and D_53_ are mutually exclusive [25], strongly suggesting that CovR has three possible states (Figure 8D: D_53_^P^ T_65_ = active; D_53_ T_65_ = inactive I dephosphorylated; D_53_ T_65_^P^ = inactive II). It should be noted that Stk1 has additional direct targets beyond CovR and that its activity depends upon its associated phosphatase Stp1 [66]–[69]. Interestingly, a Δ*stk1* mutant is affected in cell cycle progression ([66] and data not shown), as observed in *S. pneumoniae* and other bacteria [70], [71], and Stk1 might respond to the release of cell wall components into the extracellular environment by growing bacteria [72]. Therefore, it appears that CovR integrates at least two types of signals to coordinate virulence gene expression (*via* Abx1-CovS) with the control of bacterial division (through Stk1).

This integrated system might be necessary to accurately control GBS lifestyle from commensalism to invasive infection [15]. The attenuated virulence of both deletion and overexpression *abx1* mutants supports this dynamic view of the infectious process. The survival of animals challenged intraperitoneally with the overexpressing *abx1* mutant is reminiscent of the decreased virulence of Δ*covR* mutants previously described [11]–[13]. Several virulence genes controlled by CovR are involved at several steps of the infectious process, ranging from stress resistance to adherence to host cell [10], [11], [13], [16], [18], [73]. Among the genes directly regulated by CovR, the hemolysin/cytotoxin CylE has key functions [39]. At sub-lytic concentrations, CylE induces an anti-inflammatory response [74], while high CylE expression induces a pro-inflammatory response [41]. The inappropriate activation of a strong pro-inflammatory response during initial steps of the infectious process might explain, at least in part, the loss of virulence of mutants overexpressing *abx1* or inactivated for *covR*. *In contrario*, at a latter stage of infection, these mutants can be more virulent due to the cytotoxic effect of CylE on blood cells, as seen with a Δ*covR* mutant injected intravenously in mice [15]. Overall, these results indicate that the Abx1-CovS-CovR-Stk1 regulatory network controls the fine-tuning of virulence gene expression which is critical for GBS disease progression.

### The bacterial Abi-domain protein family

The demonstration that Abx1 is a cellular regulator of CovS represents a new function for a bacterial Abi-domain protein. The Abi-domain (Pfam02517) of Abx1 is the characteristic domain of a large transmembrane protein family (>5,000 sequences) with up to 15 members per bacterial species [35], [36]. This family is mostly uncharacterized to date in prokaryotes but includes the eukaryotic type II CaaX proteases involved in protein prenylation [36], [45]. However, prenylation appears restricted to eukaryotes and the two examples of prenylated bacterial proteins, SifA of *Salmonella typhimurium* and AnkB of *Legionella pneumophila*, are bacterial effectors modified by the eukaryotic enzymes after their injection into the host cell [75]–[77]. Our characterization of targeted mutations (Figure 2) did not sustain our initial hypothesis of a prenylation-like modification of the CylE hemolysin. Moreover, the putative catalytic residues inferred from similarities with CaaX proteases are not necessary for Abx1 activity. Given the high conservation of these residues (Figure S1), we cannot rule out that Abx proteins have protease activity. However, the main function of Abx1 as a regulator of the CovS histidine kinase does not require this putative protease activity.. Strikingly, the absence of a protease activity was already suggested for a *Staphylococcus aureus* Abi-protein involved in lysostaphin resistance [78]. Interestingly, three of the four *S. aureus* Abi-domain genes were identified recently in a genome-wide screen for altered targeting of cell-surface proteins [79]. These defects are mainly due to decreased transcription of the corresponding genes in the absence of the Abi-domain proteins [79], suggesting a conserved mechanism in gene transcriptional regulation.

Finally, it should be mentioned that about one-fifth (>1,000) of bacterial Abi-domain proteins are annotated as “abortive infection protein” due to a historical miss-annotation [36] leading to confusion between Abi phenotypes [80] and Abi-domain proteins [36]. It was also suggested that Abi proteins are involved in self-immunity against bacteriocins [81]. However, only a small subset of Abi-proteins (less than 1%) is localized in putative bacteriocin-encoding loci [81] or in operons encoding small toxins [82], [83]. In particular, the gene encoding the Abi-domain protein, SagE is located in the operon directing synthesis of the *S. pyogenes* ß-hemolysin known as streptolysin S or SLS [8], [84]. However, genetic analysis of this locus did not provide any conclusive clues about the SagE function [84]–[86]. Abi-domain proteins localized in toxin operons remain to be experimentally characterized for their involvement in toxin production and/or regulation.

### Conclusions

Studies of TCS signaling in cellular context have identified a great variety of additional TCS partners, targeting either the HK or the RR [31], [32]. In this study, we demonstrated that the Abi-domain protein Abx1 is a regulator of the HK CovS in GBS. The CovSR system was presumed to be a linear signaling pathway. We show here that the CovSR TCS is the core of a signaling network and propose a new regulatory model, depicted in Figure 8, which includes Abx1-dependent CovS regulation and Stk1-dependent CovR regulation [25], [34]. Deciphering the additional CovSR input/output associated with these cellular regulators might prove useful for understanding the GBS transition from commensalism to virulence. Strikingly, Abx1 belongs to a very large family of multi-spanning transmembrane proteins that remain mostly uncharacterized to date [35], [36] and are highly conserved at the species level. It is therefore tempting to extend our findings to other members of this family. The characterization of other Abx-like proteins in GBS (Figure S1) and in other bacteria will reveal whether they belong to a functionally conserved family of HK regulators.

## Materials and Methods

### Ethics statement

Animal experiments were performed at the Institut Pasteur (Paris, France) animal husbandries in accordance with the policies of the European Union guidelines for the handling of laboratory animals (http://ec.europa.eu/environment/chemicals/lab_animals/home_en.htm) and were approved by the Institut Pasteur animal care and use committee (N°04.118).

### Bacterial strains and growth conditions

The reference WT GBS strain used in this study is NEM316, a ST-23 and serotype III clinical isolate responsible for a fatal case of septicaemia, whose sequenced genome [43] is accessible (NCBI RefSeq NC_004368.1). The relevant characteristics of the bacterial strains and plasmids used in this study are summarized in Table S2. GBS was cultured in Todd Hewitt (TH) broth (Difco Laboratories) at 37°C without agitation. *Escherichia coli* DH5α (Invitrogen) and XL1 Blue (Stratagene) were grown in Luriani Broth (LB) medium. When specified, antibiotics were used at the following concentrations: for *E. coli*: ticarcillin, 100 µg/ml; erythromycin, 150 µg/ml; kanamycin, 25 µg/ml; for GBS: erythromycin, 10 µg/ml; kanamycin, 1,000 µg/ml.

### Characterization of pigmentation and hemolytic activity

Pigmentation and ß-hemolytic activity were detected on Granada agar and Columbia agar supplemented with 5% horse blood, respectively (BioMérieux, France). Overnight GBS cultures in TH were washed, adjusted to 10^8^ CFU/ml, serially diluted (dilution factor 10) in microplates and spotted on appropriate agar plates. Horse blood plates were incubated at 37°C for 16–24 h, followed by an additional 16–24 h incubation at room temperature or 4°C depending on the hyper- or hypo-hemolytic mutants being tested. To highlight the halo of lysis due to the ß-hemolysin, photographic acquisitions of blood agar plates were taken with light from below. Whole images were further converted to gray scale and processed (Photoshop CS4, Adobe, US) to adjust contrast and brightness. Granada plates were incubated 20–36 h at 37°C in anaerobic condition (AnaeroGen, Oxoid, UK). Photographic acquisitions of TH and Granada plates were done with side lighting from above and whole images were processed for contrast and brightness.

GBS hemolytic titers were determined by a semi-quantitative method as described previously, with slight modifications [11]. Briefly, culture adjusted to 2.10^8^ CFU/ml in Phosphate Buffer Saline (PBS) solution supplemented with 0.2% glucose were serially diluted (dilution factor = 2) in microplates. One volume (100 µl) of 1% defibrinated horse blood (Oxoid, UK) in 0.2% glucose PBS was added in each well and plates were incubated 1 h at 37°C. After gentle centrifugation (5 min. at 1000 rpm) to pellet unlysed cells, the amount of hemoglobin in 100 µl of supernatants was quantified by optical absorbance at 420 nm. Hemolytic activity of each strain was defined as the minimum dilution that lysed at least 10% of red blood cells. Hemolytic titers were defined by the ratio between hemolytic activities of each strain against the hemolytic activity of the WT strain (titer X = activity X/activity WT). Negative and positive controls were PBS ( = 0% lysed cells) and 0.1% SDS ( = 100% lysed cells) instead of bacterial cells, respectively. All assays were performed in triplicates with independent cultures.

### DNA manipulation and mutant construction

Purification of GBS genomic DNA and *E. coli* plasmids DNA was done on columns following manufacturer instructions (DNeasy Blood and Tissue kit and Quiaprep Spin Minipreps kit, respectively, Qiagen). Oligonucleotides (MWG and Sigma) used in this study are listed in Table S3. Analytical PCR used standard Taq polymerase (Invitrogen, Life technologies) and preparative PCR for cloning and sequencing was carried out with a high fidelity polymerase (Phusion, Finnzymes). Sanger sequencing was done at the Institut Pasteur sequencing core facility (Paris, France) using the ABI PRISM 3.1 dye terminator cycle sequencing kit (Applied Biosystems) or outsourced (Beckman Coulter Genomic, UK).

Plasmids for overexpression and complementation (pTCV backbone, Table S2), deletion (pG+host5 backbone, Table S2) and for bacterial double hybrid (pUT18 and pKNT25 backbones, Table S2) were constructed by standard cloning procedures and all inserts were fully sequenced. Primer pairs, DNA matrix and restriction enzymes used for each vector are detailed in Table S4. For several constructs, we used a splicing by overlap-extension method [87] to generate in-frame deletion cassettes, site-directed mutagenesis inserts or translational fusions (Table S4). For instance, deletion cassettes for chromosomal in-frame deletions were generated by combining two 500 bp PCR products, corresponding to genomic sequences flanking the region to be deleted, that were designed to have 25–50 bp of homology with each other at one end [87]. The 1 kb amplification products obtained with external primers were subsequently cloned into the thermosensitive shuttle plasmid pG+host5 backbone and sequenced (Table S4).

Plasmids were introduced in GBS by electroporation. For chromosomal gene inactivation/modification, allelic exchanges were selected as described [88], [89]. Deletions and site-directed substitutions were confirmed by sequencing PCR products obtained with primers designed outside the genomic region used for the construction of the corresponding cassette (Table S3 and S4).

### Detection of cell wall and secreted proteins

Secreted proteins were purified from 40 ml TH broth cultures collected at mid-exponential phase (DO_600 nm_ = 0.5). Supernatants were filter sterilized and concentrated 50-fold using Vivaspin 20 columns (Sartorius). Cell-wall proteins were prepared from overnight cultures at 37°C in TH by mutanolysin (Sigma-Aldrich) digestion in osmo-protective buffer as described [73], [89]. Following SDS-PAGE electrophoresis, proteins were transferred onto a nitrocellulose membrane (GE Healthcare) and detected using rabbit specific polyclonal antibodies [73] and horseradish peroxidase (HRP)-coupled anti-rabbit secondary antibodies. Signals were detected by chemiluminescence (ECL, GE Healthcare).

### RNA isolation and analysis

Total RNAs were extracted from exponentially growing cells (OD_600_ = 0.4–0.5) in TH at 37°C with a phenol/Trizol-based purification method as previously described [11]. Reverse transcription was done with Superscript indirect cDNA kit (Invitrogen, Life technologies) and qPCR was carried out with SYBR Green PCR kits (Applied Biosystems, Life technologies). Relative quantification of specific gene expression was calculated with the 2^−ΔΔCt^ method, with *gyrA* as the housekeeping reference, and normalized against the NEM316 wild-type. Each assay was performed at least in triplicate on three independent cultures.

For microarray analysis, identical culture conditions and RNA purification procedures were used. The three independent cDNA preparations of each strain were labeled with Cy5 or Cy3 (Amersham Biosciences) for dye swap hybridizations against NEM316 wild-type strain RNA prepared in parallel. The 15K custom microarray (Agilent Technologies) contains 8,691 60-mer oligonucleotides specific for the 2134 predicted genes and long intergenic region of the NEM316 strain [90]. Arrays were scanned in an Axon 4000B dual laser scanner and treated as described [90]. Raw data have been submitted to the ArrayExpress database under the Accession number E-MEXP-3703 (http://www.ebi.ac.uk/arrayexpress/). Hierarchical clustering were done with Cluster 3.0 and visualized with Java TreeView.

### Bacterial two-hybrid (BATCH) system

For BACTH assays [48], chimeric GBS proteins fused to either T18 or T25 fragments of adenylate cyclase (CyaA) were constructed by conventional cloning in pUT18 and pKNT25 vectors for T18/25 C-terminal tagging or in pUT18C and pKT25 for T18/25 N-terminal tagging (Table S2 and S4). Physical interactions were assayed in *E. coli* DHT1 cells (Table S2) co-transformed with recombinant pKNT25/pKT25 and pUT18/pUT18C vectors. Interaction efficiencies between hybrid proteins were quantified by measuring ß-galactosidase activity in 96-well plates assays. Bacteria were grown overnight at 30°C in 1 ml LB broth in the presence of 0.5 mM IPTG and appropriate antibiotics in 2.2 ml 96-deepwell plates (Thermo Scientific). To permeabilize cells, 100 µl of bacterial cultures in 96 deepwell polypropylene plates were treated with 500 µl of buffer containing ß-mercaptoethanol (50 mM), SDS (0.2% v/v) and chloroform (3% v/v) and vortex two times 1 min (Mixmate, Eppendorf). For the enzymatic reaction, 40 µl of ONPG (4 mg/ml) was added to 120 µl of the permeabilized cells solution in a new microplate. Reaction kinetics at 28°C were followed by recording the OD_420_ every 4 min for 60–90 min in a microplate reader (Synergy, BioTek). Slopes (min^−1^) with a correlation coefficient r^2^≥0.98 were used to calculated enzymatic activities relative to internal negative (pUT18 and pKNT25 empty vectors; activity = O arbitrary units) and positive controls (pUT18-zip and pKNT25-zip vectors, [48]; activity = 1,000 arbitrary units) incorporated in each microplate. Slopes_420_ was divided by the OD_600_ of each culture to normalize for initial cell density. At least four independent cultures were done for each plasmid combination. All recombinant pKNT25/pKT25 and pUT18/pUT18C vectors were tested against the corresponding empty vector and gave background level of ß-galactosidase activity (not depicted in Figure 5).

### *In vivo* virulence assay

Two-days old neonatal Sprague-Dawley rat pups (Janvier, Le Genest Saint Isle, France) were used for mortality curve experiments. Randomized groups of 10 neonatal rat pups were infected by intraperitoneal (i.p.) injection with 5×10^6^ bacteria in 100 µl PBS. Survivals were monitored for five days after injection and two independent experiments were carried out.

## Supporting Information

Figure S1 **Abx1 is a multi-spanning transmembrane protein belonging to a large family related to CaaX prenyl proteases.** (A) Prediction of transmembrane domains in the Abx1 sequence by the TMHMM v.2.0 software (Center for Biological Sequence Analysis, Technical University of Denmark). The eight predicted transmembrane domains with a probability greater than 0.5 are illustrated above with red boxes. The four transmembrane domains containing the conserved Abi-domain signature are highlighted with a yellow box. (B) Identification of Abi-domain proteins in the genome of *S. agalactiae*, *S. pneumoniae*, and *S. pyogenes*. Proteins containing the Abi-domain (Pfam domain PF02517, Wellcome Trust Sanger Institute, UK) were retrieved from a representative genome of each species. Protein length, Abi-domain start and end residues used for the alignment, and Abi-domain score and probabilities are given for each protein of this family. For comparaison, two eukaryotic CaaX proteases from *S. cerevisiae* (ScRCE1) and *H. sapiens* (HsBRCA2) are included. (C) Cladogram of selected streptococcal Abi-domain proteins. Multiple alignments of full protein sequences and unrooted tree were done with ClustalX 2.1 (University College Dublin, Ireland). Tree branches with a bootstrap value above >0.9 (1,000 repetitions) are indicated with a red diamond. The dark-red diamond represents a bootstrap value above >0.9 when analysing only the Abi-domain of each proteins as represent in (D). *S. agalactiae*, *S. pneumoniae*, and *S. pyogenes* proteins are in blue, red and green letters, respectively. (D) Multiple sequence alignments of streptococcal Abi-domain adapted with the representation of Pei J., Grishin N.V. and co-workers [35], [36]. The conserved topology included the four predicted transmembrane domains (TMH1 to TMH4) that are mainly made of uncharged residues (yellow background) and each containing a typical motif (motif 1 to motif 4). Highly conserved residues of motifs 1 (EExxxR), 2 (FxxxH) and 4 (HxxxN) are shown in white letters on a black background, while substitutions at these sites are on gray background. Putative cation-binding residues (1 glutamyl, E; 2 histidyl, H) and the predicted catalytic site (1 glutamyl) are marked below the two eukaryotic CaaX prenyl proteases with an asterisk and a plus sign, respectively. Seryl (S) and threonyl (T) residues in THM2 are highlighted with a green background. The more variable motif 3 (sxxxs, where s = small residues) is highlighted with a violet background. This variable motif is useful to classify Abi-domain proteins when related protein super-families (not depicted here) are considered [36].
(TIF)

Figure S2 **Conservation of the Abx1 function at the species level.** Deletion (A) and overexpression (B) of *abx1* were carried out in the WT strains BM110 (serotype III, ST17), 2603 V/R (serotype V, ST110), 515 (Serotype Ia, ST23), H36B (Serotype Ib, ST6) and 18RS21 (Serotype II, ST19). The *abx1* gene is conserved among the core genome of WT GBS isolates. It is 100% identical in the NEM316, 2603 V/R, H36B, and 18RS21 genomes; and differs by 1 SNP in the 515 genome (resulting in one amino acid change T_107_I); and by 5 SNPs in the BM110 genome (all being silent mutation at the protein level). Deletion mutant (Δ*abx1*) and return to the *abx1* WT allele (WT_back_) were selected after chromosomal integration of the *abx1* deletion vector in each strain and the subsequent chromosomal excision of this vector. For *abx1* overexpression, each strain was transformed with the pTCV empty vector and the pTCVΩP_cyl+__*abx1* overexpressing vector (abbreviated P_cyl+__*abx1*). Serial dilutions (10 fold factor) of cultures were spotted on TH, Granada and Columbia supplemented with 5% horse blood agar plates. Erythromycine (10 µg/ml) was added when necessary for plasmids maintenance. Plates were photographed after 16–36 h of growth.
(TIF)

Figure S3 **Transcriptomes profiling of** ***abx1*** **and** ***covS/R*** **mutants.** (A) Heatmap of the genes (N = 688) with an absolute log_2_ ratio >1 in at leat one strain. Deletion (Δ) for *abx1*, *covS*, *covR* and the double *covRS* mutants were compared to the *abx1* over-expression (Oe) mutants obtained by chromosomal (K_) substitution of the endogenous promoter or with the over-expression vector (V_; plasmid pTCVΩP_cyl+_ *_abx1*). The WT strain with the empty vector (NEM_ery) was added to take into account the effect of the selection pressure necessary for plasmid stability (erythromycin 10 µg/ml). Hierarchical clustering (uncentered; average linkage) was applied for genes (upper tree) and for arrays/strains (right tree). Gene expression changes were color-coded (blue = down; yellow = up). Main genes clusters are highlighted with red stars on gene tree and red boxes below the heatmaps. A short description of the main characteristic of each cluster (number 1 to 8) is given. (B) Highlight of the gene cluster 1 in (A) containing the genes negatively regulated by CovR conserved in 4 different GBS serotypes, as defined by independent groups [13], [15]. Gene tree is shown on the left of the heatmap and the corresponding systematic names and short annotations are on the right. The conserved CovR-regulated genes are in dark red letters.
(TIF)

Table S1 **Transcriptome analyses of** ***abx1*** **and** ***cov*** **mutants.** (A) Normalized expression ratio for 1905 genes. (B) Number of genes differentially regulated. (C) Definition of the CovR regulon in NEM316 and A909 WT starins. (D) List of 229 genes excluded from the analysis.
(XLSX)

Table S2 **Bacterial strains and plasmids.**
(PDF)

Table S3 **Primer sequences.**
(PDF)

Table S4 **Plasmid construction.**
(PDF)
