# Supplementary figures and images for: The Abi-domain Protein Abx1 Interacts with the CovS Histidine Kinase to Control Virulence Gene Expression in Group B Streptococcus
Source: PLoS Pathog. 2013 Feb 21;9(2):e1003179. doi: 10.1371/journal.ppat.1003179 (PMC3578759; doi:10.1371/journal.ppat.1003179)

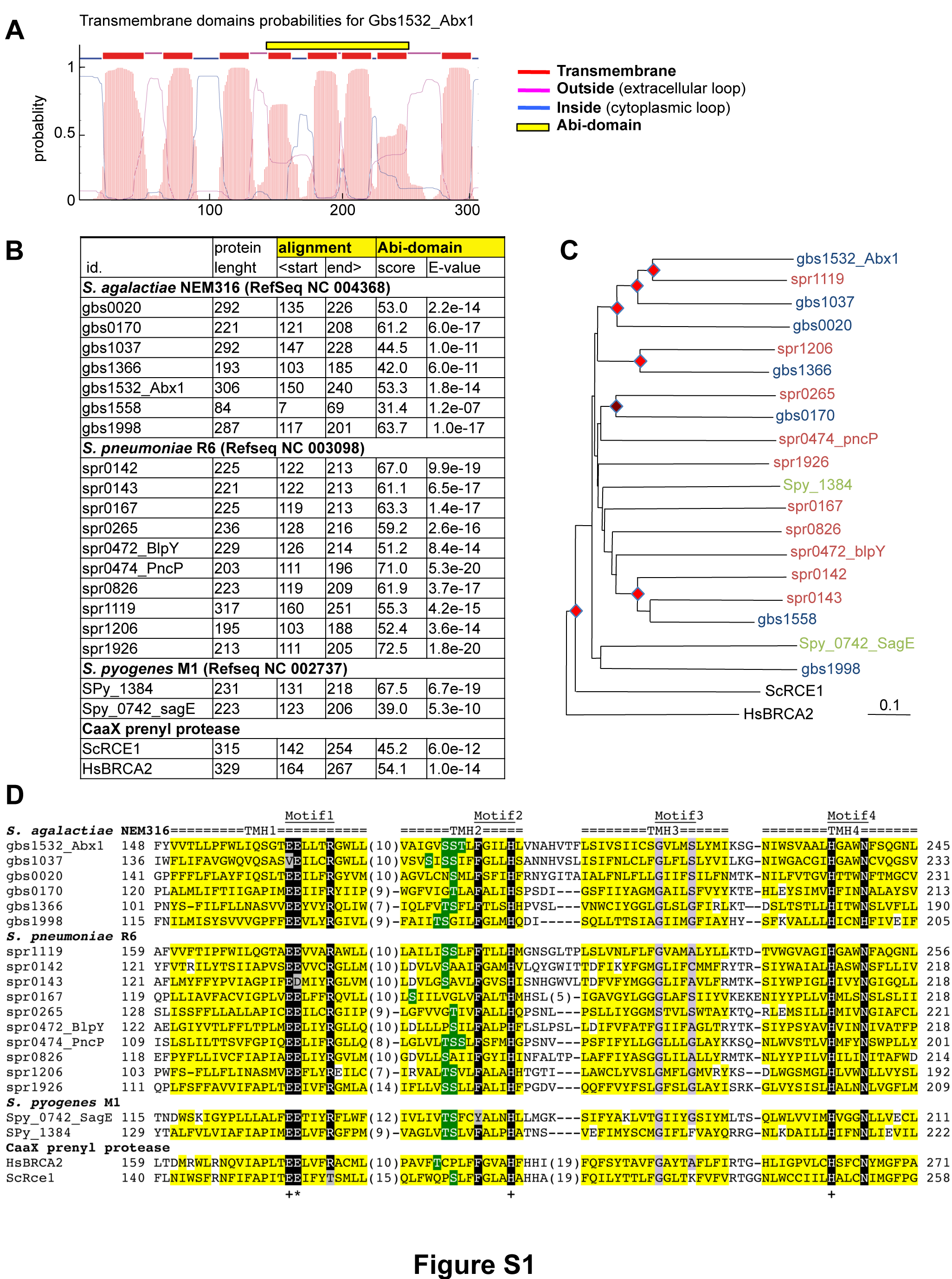

Supplement: Figure S1 — Abx1 is a multi-spanning transmembrane protein belonging to a large family related to CaaX prenyl proteases. (A) Prediction of transmembrane domains in the Abx1 sequence by the TMHMM v.2.0 software (Center for Biological Sequence Analysis, Technical University of Denmark). The eight predicted transmembrane domains with a probability greater than 0.5 are illustrated above with red boxes. The four transmembrane domains containing the conserved Abi-domain signature are highlighted with a yellow box. (B) Identification of Abi-domain proteins in the genome of S. agalactiae, S. pneumoniae, and S. pyogenes. Proteins containing the Abi-domain (Pfam domain PF02517, Wellcome Trust Sanger Institute, UK) were retrieved from a representative genome of each species. Protein length, Abi-domain start and end residues used for the alignment, and Abi-domain score and probabilities are given for each protein of this family. For comparaison, two eukaryotic CaaX proteases from S. cerevisiae (ScRCE1) and H. sapiens (HsBRCA2) are included. (C) Cladogram of selected streptococcal Abi-domain proteins. Multiple alignments of full protein sequences and unrooted tree were done with ClustalX 2.1 (University College Dublin, Ireland). Tree branches with a bootstrap value above >0.9 (1,000 repetitions) are indicated with a red diamond. The dark-red diamond represents a bootstrap value above >0.9 when analysing only the Abi-domain of each proteins as represent in (D). S. agalactiae, S. pneumoniae, and S. pyogenes proteins are in blue, red and green letters, respectively. (D) Multiple sequence alignments of streptococcal Abi-domain adapted with the representation of Pei J., Grishin N.V. and co-workers [35], [36]. The conserved topology included the four predicted transmembrane domains (TMH1 to TMH4) that are mainly made of uncharged residues (yellow background) and each containing a typical motif (motif 1 to motif 4). Highly conserved residues of motifs 1 (EExxxR), 2 (FxxxH) and 4 (HxxxN) are shown [file ppat.1003179.s001.tif]

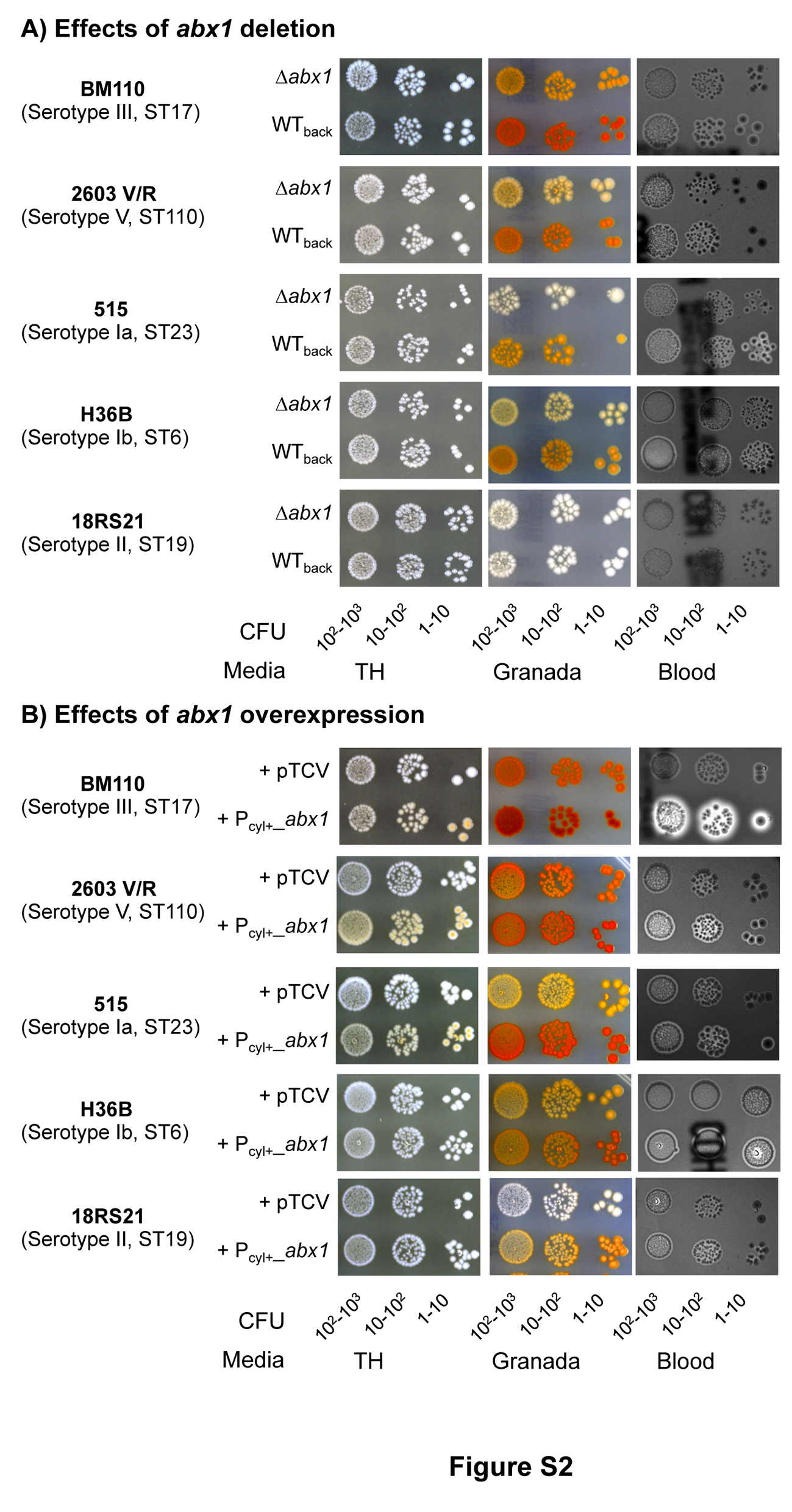

Supplement: Figure S2 — Conservation of the Abx1 function at the species level. Deletion (A) and overexpression (B) of abx1 were carried out in the WT strains BM110 (serotype III, ST17), 2603 V/R (serotype V, ST110), 515 (Serotype Ia, ST23), H36B (Serotype Ib, ST6) and 18RS21 (Serotype II, ST19). The abx1 gene is conserved among the core genome of WT GBS isolates. It is 100% identical in the NEM316, 2603 V/R, H36B, and 18RS21 genomes; and differs by 1 SNP in the 515 genome (resulting in one amino acid change T107I); and by 5 SNPs in the BM110 genome (all being silent mutation at the protein level). Deletion mutant (Δabx1) and return to the abx1 WT allele (WTback) were selected after chromosomal integration of the abx1 deletion vector in each strain and the subsequent chromosomal excision of this vector. For abx1 overexpression, each strain was transformed with the pTCV empty vector and the pTCVΩPcyl+_abx1 overexpressing vector (abbreviated Pcyl+_abx1). Serial dilutions (10 fold factor) of cultures were spotted on TH, Granada and Columbia supplemented with 5% horse blood agar plates. Erythromycine (10 µg/ml) was added when necessary for plasmids maintenance. Plates were photographed after 16–36 h of growth. (TIF) [file ppat.1003179.s002.tif]

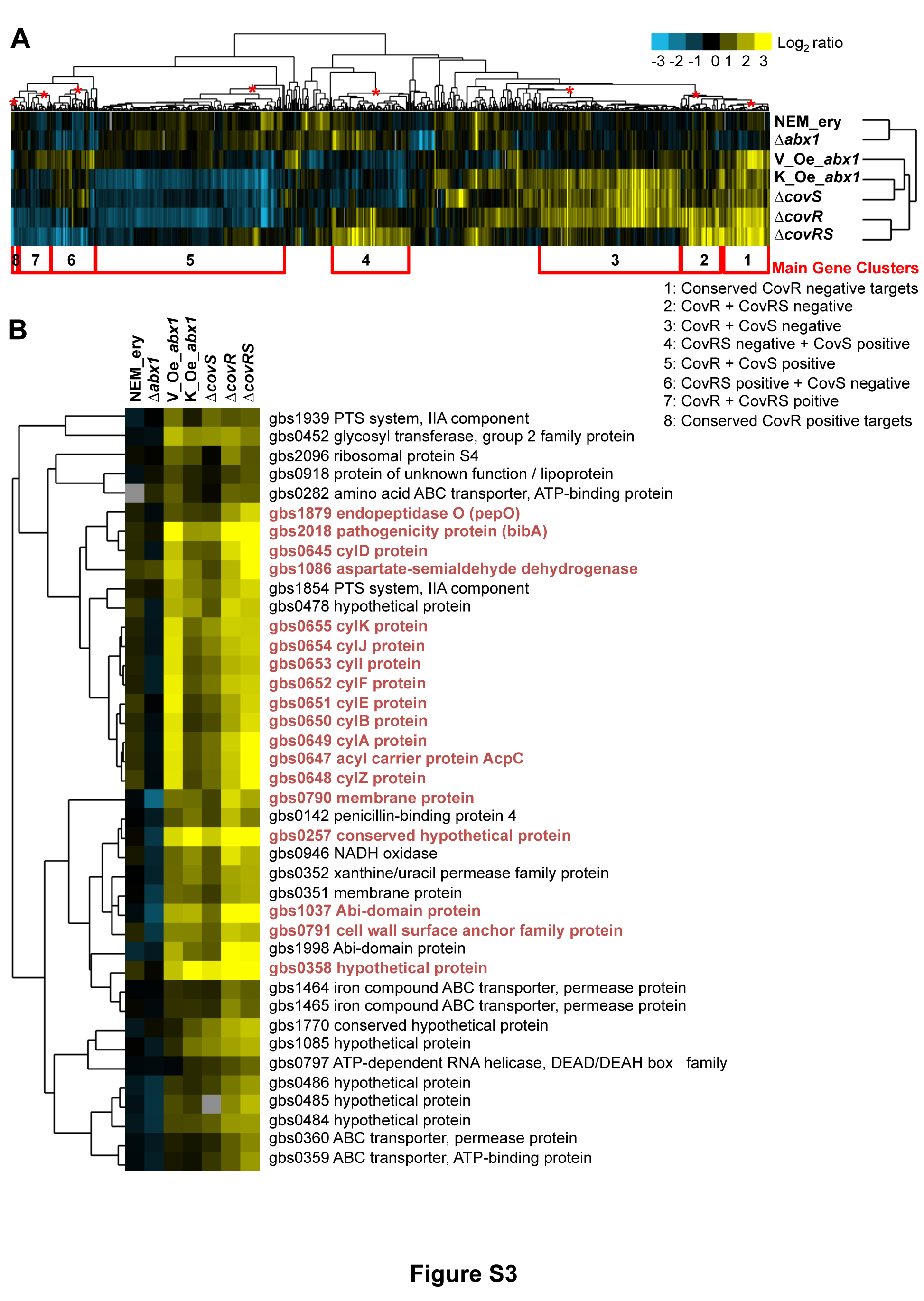

Supplement: Figure S3 — Transcriptomes profiling of abx1 and covS/R mutants. (A) Heatmap of the genes (N = 688) with an absolute log2 ratio >1 in at leat one strain. Deletion (Δ) for abx1, covS, covR and the double covRS mutants were compared to the abx1 over-expression (Oe) mutants obtained by chromosomal (K_) substitution of the endogenous promoter or with the over-expression vector (V_; plasmid pTCVΩPcyl+ _abx1). The WT strain with the empty vector (NEM_ery) was added to take into account the effect of the selection pressure necessary for plasmid stability (erythromycin 10 µg/ml). Hierarchical clustering (uncentered; average linkage) was applied for genes (upper tree) and for arrays/strains (right tree). Gene expression changes were color-coded (blue = down; yellow = up). Main genes clusters are highlighted with red stars on gene tree and red boxes below the heatmaps. A short description of the main characteristic of each cluster (number 1 to 8) is given. (B) Highlight of the gene cluster 1 in (A) containing the genes negatively regulated by CovR conserved in 4 different GBS serotypes, as defined by independent groups [13], [15]. Gene tree is shown on the left of the heatmap and the corresponding systematic names and short annotations are on the right. The conserved CovR-regulated genes are in dark red letters. (TIF) [file ppat.1003179.s003.tif]
