# Supplementary material for: The Abi-domain Protein Abx1 Interacts with the CovS Histidine Kinase to Control Virulence Gene Expression in Group B Streptococcus
Source: PLoS Pathog. 2013 Feb 21;9(2):e1003179. doi: 10.1371/journal.ppat.1003179 (PMC3578759; doi:10.1371/journal.ppat.1003179)
Supplement: Table S2 — Bacterial strains and plasmids. (PDF) [file ppat.1003179.s005.pdf]

Supplementary Table S2: Bacterial strains and plasmids.

Streptococcus agalactiae

Strain	Relevant genotype	Plasmid	Reference
NEM316	Clinical isolate, Serotype III		[1]
NEM2717	NEM316	pTCV-*erm*	This study
NEM2716	NEM316	pTCVΩ*abx1*	This study
NEM2713	NEM316 *gbs1532*::*Him1*	pTCV-*erm*	This study
NEM2712	NEM316 *gbs1532*::*Him1*	pTCVΩ*abx1*	This study
NEM2715	NEM316 *gbs1532*::*Him2*	pTCV-*erm*	This study
NEM2714	NEM316 *gbs1532*::*Him2*	pTCVΩ*abx1*	This study
NEM2782	NEM316 Δ*abx1*		This study
NEM2787	NEM316 Δ*abx1*	pTCV-*erm*	This study
NEM2788	NEM316 Δ*abx1*	pTCVΩ*abx1*	This study
NEM2857	NEM316	pTCVΩP$_{cyl+}$_*abx1*	This study
NEM3007	NEM316	pTCVΩP$_{tet}$_*abx1*	This study
NEM3003	NEM316	pTCVΩP$_{cyl+}$_*gbs1037*	This study
NEM3006	NEM316	pTCVΩP$_{cyl+}$_*EGFP*	This study
NEM3010	NEM316 ΔP$_{abx1}$ ::P$_{cyl+}$		This study
NEM2456	NEM316 Δ*cylE*		[2]
NEM2948	NEM316 Δ*cylE*	pTCV-*erm*	This study
NEM2949	NEM316 Δ*cylE*	pTCVΩP$_{cyl+}$_*abx1*	This study
NEM2722	NEM316 *CylE* C$_{664}$A		This study
NEM2960	NEM316 Δ*abx1*	pTCVΩ*abx1*_E$_{164}$A	This study
NEM2961	NEM316 Δ*abx1*	pTCVΩ*abx1*_E$_{165}$A	This study
NEM2962	NEM316 Δ*abx1*	pTCVΩ*abx1*_EE$_{164/165}$AA	This study
NEM2860	NEM316 Δ*abx1*	pTCVΩ*abx1*_H$_{197}$A	This study
NEM2861	NEM316 Δ*abx1*	pTCVΩ*abx1*_H$_{235}$A	This study
NEM2802	NEM316 Δ*covR*		This study
NEM2089	NEM316 Δ*covSR*		[3]
CCH206	NEM316 ΔCBS$_{cyl}$		[2]
NEM2810	NEM316 Δ*covR* Δ*abx1*		This study
NEM2811	NEM316 Δ*covSR* Δ*abx1*		This study
NEM2783	NEM316 ΔCBS$_{cyl}$ Δ*abx1*		This study
CCH375	NEM316 Δ*bibA*		[4]
NEM2582	NEM316 Δ*camp*		[5]
NEM2803	NEM316 Δ*covS*		This study
NEM3211	NEM316 *CovS* H$_{278}$A		This study
NEM3300	NEM316 *CovS* T$_{282}$A		This study
NEM2899	NEM316 *CovR* D$_{53}$A		This study
NEM2809	NEM316 Δ*covS* Δ*abx1*		This study
NEM2999	NEM316 Δ*covS*	pTCVΩP$_{cyl+}$_*abx1*	This study
NEM3219	NEM316 *CovS* H$_{278}$A	pTCVΩP$_{cyl+}$_*abx1*	This study
NEM3324	NEM316 *CovS* T$_{282}$A	pTCVΩP$_{cyl+}$_*abx1*	This study
NEM3327	NEM316 (*CovS* H$_{278}$A)-*CovS*$_{wt}$		This study
NEM3329	NEM316 (*CovS* T$_{282}$A)-*CovS*$_{wt}$		This study
NEM3339	NEM316 (*CovS* H$_{278}$A)-*CovS*$_{wt}$	pTCVΩP$_{cyl+}$_*abx1*	This study
NEM3342	NEM316 (*CovS* T$_{282}$A)-*CovS*$_{wt}$	pTCVΩP$_{cyl+}$_*abx1*	This study
NEM2698	NEM316 Δ*stk1*		This study
NEM2812	NEM316 Δ*stk1*	pTCV-*erm*	This study
NEM2936	NEM316 Δ*stk1*	pTCVΩ*stp_stk*	This study
NEM2813	NEM316 Δ*stk1*	pTCVΩ*abx1*	This study
NEM2937	NEM316 Δ*stk1*	pTCVΩP$_{cyl+}$_*abx1*	This study
NEM2699	NEM316 Δ*stk1* Δ*covS*		This study
NEM3072	NEM316 Δ*stk1* Δ*covS*	pTCV-*erm*	This study
NEM3073	NEM316 Δ*stk1* Δ*covS*	pTCVΩ*abx1*	This study
NEM3074	NEM316 Δ*stk1* Δ*covS*	pTCVΩP$_{cyl+}$_*abx1*	This study
BM110	Clinical isolate, Serotype III		[4]
2603 V/R	Clinical isolate, Serotype V		[6]

515	Clinical isolate, Serotype Ia		[7]
H36B	Clinical isolate, Serotype Ib		[7]
18RS21	Clinical isolate, Serotype II		[7]
NEM3627	BM110 Δ*abx1*		This study
NEM3628	BM110 WT_{back}		This study
NEM3600	2603 V/R Δ*abx1*		This study
NEM3601	2603 V/R WT_{back}		This study
NEM3604	515 Δ*abx1*		This study
NEM3605	515 WT_{back}		This study
NEM3608	H36B Δ*abx1*		This study
NEM3609	H36B WT_{back}		This study
NEM3610	18RS21 Δ*abx1*		This study
NEM3611	18RS21 WT_{back}		This study
NEM3631	BM110	pTCV-*erm*	This study
NEM3244	2603V/R	pTCV-*erm*	This study
NEM3246	515	pTCV-*erm*	This study
NEM3247	H36B	pTCV-*erm*	This study
NEM3248	18RS21	pTCV-*erm*	This study
NEM3632	BM110	pTCVΩP_{cyl+}_*abx1*	This study
NEM3249	2603V/R	pTCVΩP_{cyl+}_*abx1*	This study
NEM3251	515	pTCVΩP_{cyl+}_*abx1*	This study
NEM3252	H36B	pTCVΩP_{cyl+}_*abx1*	This study
NEM3253	18RS21	pTCVΩP_{cyl+}_*abx1*	This study

Escherichia coli

Strain	Genotype	Reference
DHT1	F^- *glnV44*(AS) *recA1 endA1 gyrA96* (Nal^r) *thi-1 hsdR17 spoT1 rfbD1 cya-854 ilv-691* ::Tn*10*	[8]
DH5∝	F^- *recA1 endA1 gyrA96 thi-1 relA1* Δ(*lacIZYA-argF*)[ϕ*80*Δ*lacZ*Δ*M15*] *hsdR17* glnV44 *deoR nupG*	Invitrogen
XL1-Blue	*endA1 gyrA96* (nal^R) *thi-1 recA1 relA1 lac glnV44* F'[::*Tn10 proAB*$^+$ *lacI*^{q} Δ(l*acZ*)*M15*] *hsdR17*(r_K^- m_K^+)	Stratagene

Plasmids 1: plasmids published

Plasmid	Resistance markers	Relevant properties	Reference
pG$^+$host5	Erythromycin	ColE1 replicon, thermosensitive derivative of pGK12	[9]
pTCV-*erm*	Erythromycin Kanamycin	Mob$^+$ (IncP); *oriR* pACYC184; *oriR* pAM_1	[10]
pKNT25	Kanamycin	BATCH vector, T25 fragment at the C-terminal end of the fusion protein, p15 ori	[11]
pUT18	Ampicilin	BATCH vector, T18 fragment at the C-terminal end of the fusion protein, ColE1 ori	[11]
pKT25	Kanamycin	BATCH vector, T25 fragment at the N-terminal end of the fusion protein, p15 ori	[12]
pUT18C	Ampicilin	BATCH vector, T18 fragment at the N-terminal end of the fusion protein, ColE1 ori	[12]
pKNT25-zip	Kanamycin	BATCH vector, positive control, p15 ori	[12]
pUT18-zip	Ampicilin	BATCH vector, positive control, ColE1 ori	[12]

Plasmids 2: plasmids constructed for this study

Plasmid	Relevant characteristics
pTCVΩ*abx1*	*abx1* complementing vector, promoter P_{abx1}
pTCVΩP_{cyl+}_*abx1*	*abx1* overexpression vector, promoter P_{cyl+}
pTCVΩP_{tet}_*abx1*	*abx1* overexpression vector, promoter P_{tet}
pTCVΩP_{cyl+}_*gbs1037*	*gbs1037* overexpression vector, promoter P_{cyl+}

pTCVΩP_{cyl+}_*EGFP*	*EGFP* overexpression vector, promoter P_{cyl+}
pTCVΩ*abx1*_E_{164}A	Abx1_E_{164}A expression vector, promoter P_{abx1}
pTCVΩ*abx1*_E_{165}A	Abx1_E_{165}A expression vector, promoter P_{abx1}
pTCVΩ*abx1*_$EE_{164/165}$AA	Abx1_ $EE_{164/165}$AA expression vector, promoter P_{abx1}
pTCVΩ*abx1*_H_{197}A	Abx1_ H_{197}A expression vector, promoter P_{abx1}
pTCVΩ*abx1*_H_{235}A	Abx1_ H_{235}A expression vector, promoter P_{abx1}
pTCVΩ*stp_stk*	Stp1-Stk1 complementing vector, promoter P_{stp1}
pGΩΔ*abx1*	Vector for *abx1* in frame deletion
pGΩΔ*covR*	Vector for *covR* in frame deletion
pGΩΔ*covS*	Vector for *covS* in frame deletion
pGΩΔ*stk1*	Vector for *stk1* in frame deletion
pGΩΔP_{abx1} ::P_{cyl+}	Vector for replacing the chromosomal P_{abx1} promoter by P_{cyl+}
pGΩ*covR*_D_{53}A	Vector for CovR_D_{53}A substitution on the chromosome
pGΩ*covS*_H_{278}A	Vector for CovR_ H_{278}A substitution on the chromosome
pGΩ*covS*_T_{282}A	Vector for CovR_ T_{282}A substitution on the chromosome
pGΩ*cylE*_C_{664}A	Vector for CovR_ C_{664}A substitution on the chromosome
pKNT25Ω*abx1*	BATCH expression vector, Abx1-T25 fusion
pKNT25Ω*covS*	BATCH expression vector, CovS-T25 fusion
pKNT25Ω*covR*	BATCH expression vector, CovR-T25 fusion
pKNT25Ω*gbs2082*	BATCH expression vector, Gbs2082-T25 fusion
pKNT25Ω*gbs0430*	BATCH expression vector, Gbs0430-T25 fusion
pUT18Ω*abx1*	BATCH expression vector, Abx1-T18 fusion
pUT18Ω*covS*	BATCH expression vector, CovS-T18 fusion
pUT18Ω*covR*	BATCH expression vector, CovR-T18 fusion
pUT18Ω*gbs2082*	BATCH expression vector, Gbs2082-T18 fusion
pUT18Ω*gbs0430*	BATCH expression vector, Gbs0430-T18 fusion
pKT25Ω*abx1*	BATCH expression vector, T25-Abx1 fusion
pKT25Ω*covS (form VII)*	BATCH expression vector, T25-CovS fusion
pUT18CΩ*abx1*	BATCH expression vector, T18-Abx1 fusion
pUT18CΩ*covS (form VII)*	BATCH expression vector, T18-CovS fusion
pKNT25Ω*covS form I*	BATCH expression vector, CovS (residues 213-501)-T25 fusion
pKNT25Ω*covS form II*	BATCH expression vector, CovS (residues 186_501)-T25 fusion
pKNT25Ω*covS form III*	BATCH expression vector, CovS (residues 186_266)-T25 fusion
pKT25Ω*covS form IV*	BATCH expression vector, T25 - CovS (residues 5_213) fusion
pKT25Ω*covS form V*	BATCH expression vector, T25 - CovS (residues 5_45) fusion
pKT25Ω*covS form VI*	BATCH expression vector, T25 - CovS (minus residues 46_185) fusion
pUT18Ω*covS form I*	BATCH expression vector, CovS (residues 213-501)-T18 fusion
pUT18Ω*covS form II*	BATCH expression vector, CovS (residues 186_501)-T18 fusion
pUT18Ω*covS form III*	BATCH expression vector, CovS (residues 186_266)-T18 fusion
pUT18CΩ*covS form IV*	BATCH expression vector, T18 - CovS (residues 5_213) fusion
pUT18CΩ*covS form V*	BATCH expression vector, T18 - CovS (residues 5_45) fusion
pUT18CΩ*covS form VI*	BATCH expression vector, T18 - CovS (minus residues 46_185) fusion

Additional References for Supplementary Table S2

1. Glaser P, Rusniok C, Buchrieser C, Chevalier F, Frangeul L, et al. (2002) Genome sequence of *Streptococcus agalactiae*, a pathogen causing invasive neonatal disease. Mol Microbiol 45: 1499-1513.
2. Forquin MP, Tazi A, Rosa-Fraile M, Poyart C, Trieu-Cuot P, et al. (2007) The putative glycosyltransferase-encoding gene *cylJ* and the Group B Streptococcus (GBS)-specific gene *cylK* modulate hemolysin production and virulence of GBS. Infect Immun 75: 2063-2066.
3. Lamy MC, Zouine M, Fert J, Vergassola M, Couve E, et al. (2004) CovS/CovR of group B streptococcus: a two-component global regulatory system involved in virulence. Mol Microbiol 54: 1250-1268.
4. Tazi A, Disson O, Bellais S, Bouaboud A, Dmytruk N, et al. (2010) The surface protein HvgA mediates group B streptococcus hypervirulence and meningeal tropism in neonates. J Exp Med 207: 2313-2322.

5. Charrel-Dennis M, Latz E, Halmen KA, Trieu-Cuot P, Fitzgerald KA, et al. (2008) TLR-independent type I interferon induction in response to an extracellular bacterial pathogen via intracellular recognition of its DNA. Cell Host Microbe 4: 543-554.
6. Tettelin H, Masignani V, Cieslewicz MJ, Eisen JA, Peterson S, et al. (2002) Complete genome sequence and comparative genomic analysis of an emerging human pathogen, serotype V *Streptococcus agalactiae*. Proc Natl Acad Sci U S A 99: 12391-12396.
7. Tettelin H, Masignani V, Cieslewicz MJ, Donati C, Medini D, et al. (2005) Genome analysis of multiple pathogenic isolates of *Streptococcus agalactiae*: implications for the microbial "pan-genome". Proc Natl Acad Sci U S A 102: 13950-13955.
8. Dautin N, Karimova G, Ullmann A, Ladant D (2000) Sensitive genetic screen for protease activity based on a cyclic AMP signaling cascade in *Escherichia coli*. J Bacteriol 182: 7060-7066.
9. Biswas I, Gruss A, Ehrlich SD, Maguin E (1993) High-efficiency gene inactivation and replacement system for gram-positive bacteria. J Bacteriol 175: 3628-3635.
10. Poyart C, Lamy MC, Boumaila C, Fiedler F, Trieu-Cuot P (2001) Regulation of D-alanyl-lipoteichoic acid biosynthesis in *Streptococcus agalactiae* involves a novel two-component regulatory system. J Bacteriol 183: 6324-6334.
11. Karimova G, Dautin N, Ladant D (2005) Interaction network among *Escherichia coli* membrane proteins involved in cell division as revealed by bacterial two-hybrid analysis. J Bacteriol 187: 2233-2243.
12. Karimova G, Pidoux J, Ullmann A, Ladant D (1998) A bacterial two-hybrid system based on a reconstituted signal transduction pathway. Proc Natl Acad Sci U S A 95: 5752-5756.
