# Supplementary material for: The Abi-domain Protein Abx1 Interacts with the CovS Histidine Kinase to Control Virulence Gene Expression in Group B Streptococcus
Source: PLoS Pathog. 2013 Feb 21;9(2):e1003179. doi: 10.1371/journal.ppat.1003179 (PMC3578759; doi:10.1371/journal.ppat.1003179)
Supplement: Table S3 — Primer sequences. (PDF) [file ppat.1003179.s006.pdf]

Supplementary Table S3: Primer sequences.

Oligonucleotide	Sequence (5'-3') *	Features
For plasmids construction		
KO_*abx1_E*	AATGGAATTCGTAACAGTAGTTCCGGCCATTAGC	EcoRI
KO_*abx1*_rv	GAGCTGCAACACTCAATCTGTTTCGTTTTTATAGACAGCGAACTGACT	
KO_*abx1*_fw	AGTCAGTTCGCTGTCTATAAAAACGAAACAGATTGAGTGTTGCAGCTC	
KO_*abx1_B*	TCAAGGATCCGCTGAGTGACGTAATTTGTCAATTTCG	BamHI
O1	CTTCGAATTCTGGCAGCTGATGAGGTTGGGG	EcoRI
O2	CACATAGCCCATTCCGCGGTCCAATCCTTCACGACCG	
O3	CGCGGAATGGGCTATGTGATTCGT	
O4	CCAACGGATCCTTCCATATCTGCAACTTTAGAG	BamHI
O5	GGATATGGTACCTGGTCTTGATCGTGGAGCAG	KpnI
O6	CCCCCTGTGAAGGACGTATCTTGTTGCCGA	
O7	GTCCTTCACAGGGGGGCTGGTTTAGGTATCGGGCTC	
O8	ATACTAGGATCCTCATCTTCATACTCAC	BamHI
Stk5	ACAAAGAATTCGGAACTACCATTGAAGCAGTTGCCA	EcoRI
Stk int1	GGCACGCCCGGGTGCTGCCGCCGCCATTCCACCTCGTCCGATTGA	
Stk int2	GCGGCAGCACCCGGGCGTGCCCCAAGTGATAGTACAACTAATACAGGA	
Stk3	ACATACTGCAGTGCAAAGACAACAGCTGCAA	PstI
cylEA_E	TTGATGAATTCAGCCTTTCTTTTCCACTGAC	EcoRI
cylEA_rev	GTATTCATTTTTCTTCGGCCTTTCTATATTCTGT	
cylEA_fw	ACAGAATATAGAAAGGCCGAAGAAAAATGAATAC	
cylEA_B	TCTGCGGATCCTTCACAGACATCCGAAAC	BamHI
O9	AGTAGAATTCAAAAGTGACAGAGGACACTACTC	EcoRI
O10	TCCATCTCTGGAAGCATTAAGGCCAGTAGAATCAAATCAAAATC	
O11	GATTTTGATTTGATTCTACTGGCCTTAATGCTTCCAGAGATGGA	
O12	AGTGAAGGATCCTCATCACCACGCATCGCTGAACG	BamHI
1532_B	TCTGCGGATCCGGCGATAACTTGGCTCATTGTG	BamHI
EE_rev	AGCCACCCTCGTGTTAGCAATGCGGCCGTACCGCTTTGAATAAGCC	
EE_fw	GGCTTATTCAAAGCGGTACGGCCGCATTGCTAACACGAGGGTGGCT	
E164_rev	CACCCTCGTGTTAGCAATTCGGCCGTACCGCTTTGAATAAGC	
E164_fw	GCTTATTCAAAGCGGTACGGCCGAATTGCTAACACGAGGGTG	
E165_fw	GCTTATTCAAAGCGGTACGGAGGCCTTGCTAACACGAGGGTGGCTTC	
E165_rev	GAAGCCACCCTCGTGTTAGCAAGGCCTCCGTACCGCTTTGAATAAGC	
H197_rev	GTGACATGGGCATTTACCAAGGCCAAAATGCCAAATAAAGTGG	
H197_fw	CCACTTTATTTGGCATTTTGGCCTTGGTAAATGCCCATGTCAC	
H235_fw	GTAATATTTGGAGTGTTGCAGCCTTGGCCGGAGCTTGGAATTTTAGCCAAGG	
H235_rev	CCTTGGCTAAAATTCCAAGCTCCGGCCAAGGCTGCAACACTCCAAATATTAC	
pAF18	TAGGAATTCAGTTTCTGCACTGACCTTTCCAC	EcoRI
pAF19	TACTCTAGATGGGCGATAACTTGGCTCATTGTG	XbaI
pAF52	ACATGAATTCTTAGGCTTACTAACTTAGCT	EcoRI
pAF53	CTGACTATTTAAAATCCTTTTATTCATATTATCACCTTCAACTAATCAT	
pAF54	ATGATTAGTTGAAGGTGATAATATGAATAAAAGGATTTTAAATAGTCAG	
pAF69	ACTATCTAGATGTTCAGGTATCGGGCTAATTCGC	XbaI
pAF70	TCAGTCTAGAGGCAGAACCTTTCTAGGCATGGTC	XbaI
pAF149	GACTATTTAAAATCCTTTTATTCATACAGATATTCTCCGGATACTTT	
pAF150	AAAGTATCCGGAGAATATCTGTATGAATAAAAGGATTTTAAATAGTC	
pAF151	TCATGGATCCTTCTTCCGTACCGCTTTGAATAAGC	BamHI
pAF152	TTATGATATAATCGTTCTAGTGTTAGGCTTACTAACTTAGCTTTG	
pAF153	CAAAGCTAAGTTAGTAAGCCTAACACTAGAACGATTATATCATAA	
pAF200	CAGCTCCTCGCCCTTGCTCACCATATTATCACCTTCAACTAATCAT	
pAF201	ATGATTAGTTGAAGGTGATAATATGGTGAGCAAGGGCGAGGAGCTG	
pAF202	ATCAGGATCCGACTCTAGAGTCGCGGCCGCTTTAC	BamHI
pAF203	CCTCAAAAATAGACTCTCTCATATTATCACCTTCAACTAATCAT	
pAF204	ATGATTAGTTGAAGGTGATAATATGAGAGAGTCTATTTTTGAGG	
pAF205	AGTCGGATCCAATAAGCTTTCAGTAAGAGAAGCTT	BamHI

pAF206	TCATGAATTCGGTACTTGAAAAGAACGGGAGTAATTGG	EcoRI
pAF207	TAGTGAATTCGAAATGACCCTATACGATGTCAA	EcoRI
pAF208	CAGCGACAGGCGTTCTTAATTCGGCCGAAACATCACTAATGAATTGTGA	
pAF209	TCACAATTCATTAGTGATGTTTCGGCCGAATTAAGAACGCCTGTCGCTG	
pAF210	TCTTGGATCCAGTTCGATAAAAACGATCAAAG	BamHI
pAF216	TCATAAGCTTGAATAAAAGGATTTTAAATAGTCAG	HindIII
pAF217	TCATGAATTCGCATCTGTTTCTTCAGTCTTAATTAGC	EcoRI
pAF218	TCTACTGCAGGAAAGATCAGTTTATAGGGGTAAAAC	PstI
pAF219	TACAGGATCCGCTATTTCTTTAGTTTCTTCAAATTT	BamHI
pAF220	TCATAAGCTTGGGTAAAAAGATCTTAATAATCG	HindIII
pAF221	ATCAGAATTCGCTTTTTCACGAATCACATAGCCCATTC	EcoRI
pAF245	ACTACTGCAGGTATATTGTTATTGGTTACCTT	PstI
pAF246	ATCAGGATCCGCTTGTAATTTAAGTACTAAAGAG	BamHI
pAF234	AGCTGGATCCTTAGTAGCACTATTAATAACG	BamHI
pAF235	TAGTGGATCCGCAGTATAGTCTTCCAACTGATC	BamHI
pAF236	ATGACTGCAGGAAGCGTATTGTTCGTCCGGTTA	PstI
pAF239	TGATCTGCAGGTTCCAAAGTAAAATAAATTACC	PstI
pAF240	ACTAGGATCCTTCAATTTTGGAAAGGTGAGC	BamHI
pAF407	GACCCTTTACAACAGCGACAGGGGCCCTTAATTCATGACTAACATCAC	
pAF408	GTGATGTTAGTCATGAATTAAGGGCCCCTGTCGCTGTTGTAAAGGGTC	
pAF443	TACTGGATCCGAATAAAAGGATTTTAAATAGTCAG	BamHI
pAF444	TCTAGGTACCGCATCTGTTTCTTCAGTCTTAATTAG	KpnI
pAF245	ACTACTGCAGGTATATTGTTATTGGTTACCTT	PstI
pAF246	ATCAGGATCCGCTTGTAATTTAAGTACTAAAGAG	BamHI
pAF500	TACAGGATCCTAAAGATCAGTTTATAGGGGTAAAAC	BamHI
pAF501	GATGTTTTGCCTTGCACCCGCGTATCTTGTTGCCGAAGTATAAAC	
pAF502	CAACAAGATACGCGGGTGCAAGGCAAAACATCATTGTTTTCTTG	
pAF503	TCTAGGTACCGCTATTTCTTTAGTTTCTTCAAATTT	KpnI
For qRT-PCR		
*abx1*_qF	GTGGCTTCTTCCTCTAAT	Abx1
*abx1*_qR	CCGAACATATAATACTTACAATAG	Abx1
*cylE*_qF	TTGATTAGAGATAGTGAAGGTTA	CylE
*cylE*_qR	CCAGGAGGAGAATAGGAA	CylE
*cylJ*_qF	TAAGCAAGACAATAAGACT	CylJ
*cylJ*_qR	CTACCTCAATATAACTTCCT	CylJ
*bibA*_qF	CAACTGAATAATGCTGAATC	BibA
*bibA*_qR	AACTTGACTCTTAGTGCTA	BibA
*0791*_qF	AGTGCACAACAAGGTTTCCG	Gbs0791
*0791*_qR	ACCAATTTTCCCGGCAAGAC	Gbs0791
*1037*_qF	ATGGTTAGTCGAGAGCGTTCTC	Gbs1037
*1037*_qR	ACAGATGCGCTTTGTACCTG	Gbs1037
*CAMP*_qF	GGAACTCTAGTGGCTGGTG	CAMP
*CAMP*_qR	CATTTGCTGGGCTTGATTATTAC	CAMP
*gyrA*_qF	CCTACTGGTGCCTTGGTGATG	GyrA
*gyrA*_qR	GTGACAACAATACGCTCTTTCCC	GyrA
For sequence analysis		
KO_*abx1*_5	AGGCTGTTATTCATTAGGTCACTTG	Abx1
KO_*abx1*_3	AACCGTAAATACAAGAAACAGATGC	Abx1
O13	ATGGGTAAAAAGATCTTAATAATCGAAG	CovR
O14	AACACTGAGAGCCCGATACC	CovS
O15	GGCATCCCGTATTGCAGATG	CovS
O16	CCACGAATTCCAGATCGTATTGAGCG	CovR
O17	ACTCTGCAGCTTCATCTGAGATACCTTCTCC	CovS
cylEA_5	CCTGGCAAAGCCAGCAATCAAA	CylE
cylEA_3	TGGGTTGTCCCACTTCAAACCT	CylE
pAF48	GATTTAATGCTTCCAGAGATGGAT	CovR
pAf49	CTTTTCCCATGGATTTACCAATAG	CovS

pAF50	GGCGAGTCAATCGATAGTATTCTT	CovS
pAF51	ACACGCTACCACTCTTTTTACTCC	CovS
pAF211	AACCAGCTTGTGTACTTTCTCTG	CovS
pAF174	TATTGCTAGCGAGATGACTGTAAC	Stk1
pAF175	ATCAATTGTATCAGTCCCAGAGAT	Stk1
pAF306	AGCGACTGTTCAGAGTGACATT	Stk1
pAF230	GTTGACCAGGCGGAACATC	pKNT25
pAF231	GATATTCATGTCGCCGTCGTAG	pUT18
pAF286	GTGAGTTAGCTCACTCATTAG	pUT18
pAF308	CTTCTACGAGAACCGTGCATAC	pUT18C
pAF309	GTGAAAACCTCTGACACATGC	pUT18C
pAF310	ATCGACATGTTCGCCATTATGC	pKT25
pAF311	GATGTGCTGCAAGGCGATTAAG	pKT25

* Restriction sites are underlined
