# Supplementary material for: The Abi-domain Protein Abx1 Interacts with the CovS Histidine Kinase to Control Virulence Gene Expression in Group B Streptococcus
Source: PLoS Pathog. 2013 Feb 21;9(2):e1003179. doi: 10.1371/journal.ppat.1003179 (PMC3578759; doi:10.1371/journal.ppat.1003179)
Supplement: Table S4 — Plasmid construction. (PDF) [file ppat.1003179.s007.pdf]

Supplementary Table S4: Plasmid construction.

1st PCRs	Matrix	2nd PCR	Digestion	Vector	Analysis
Construction of pGΩΔ*abx1* for *abx1* in frame deletion					
a) KO_*abx1*_E + KO_*abx1*_rv	gDNA NEM316	KO_*abx1*_E + KO_*abx1*_B	EcoRI BamHI	pG^{+}host5	KO_*abx1*_5 KO_*abx1*_3
b) KO_*abx1*_fw + KO_*abx1*_B	gDNA NEM316				
Construction of pGΩΔ*covR* for *covR* in frame deletion					
a) O1 + O2	gDNA NEM316	O1 + O4	EcoRI	pG^{+}host5	
b) O3 + O4	gDNA NEM316		BamHI		
Construction of pGΩΔ*covS* for *covS* in frame deletion					
a) O5 + O6	gDNA NEM316	O5 + O8	KpnI	pG^{+}host5	
b) O7 + O8	gDNA NEM316		BamHI		
Construction of pGΩΔ*stk1* for *stk1* in frame deletion					
a) Stk5 + Stk int1	gDNA NEM316		EcoRI	pG^{+}host5	pAF174
b) Stk int2 + Stk3	gDNA NEM316		PstI		pAF175
Construction of pGΩΔP_{abx1}::P_{cyl+} for *abx1* promoter substitution					
a) KO_*abx1*_E + pAF152	gDNA NEM316	KO_*abx1*_E +	EcoRI	pG^{+}host5	
b) pAF153 + pAF151	pTCVΩP_{cyl+}_*abx1*	pAF151	BamHI		
Construction of pGΩ*cylE* $C_{664}A$ for CylE C_{664}A alanine substitution					
a) cylEA_E + cylEA_rev	gDNA NEM316	cylEA_E +	EcoRI	pG^{+}host5	cylEA_5
b) cylEA_fw + cylEA_B	gDNA NEM316	cylEA_B	BamHI		cylEA_3
Construction of pGΩ*covR* $D_{53}A$ for CovR D_{53}A alanine substitution					
a) O9 + O10	gDNA NEM316	O9 + O12	EcoRI	pG^{+}host5	
b) O11 + O12	gDNA NEM316		BamHI		
Construction of pGΩ*covS* $H_{278}A$ for CovS H_{278}A alanine substitution					
a) pAF207 + pAF208	gDNA NEM316	pAF207 +	EcoRI	pG^{+}host5	pAF50
b) pAF209 + pAF210	gDNA NEM316	pAF210	BamHI		pAF211
Construction of pGΩ*covS* $T_{282}A$ for CovS T_{282}A alanine substitution					
a) pAF207 + pAF407	gDNA NEM316	pAF207 +	EcoRI	pG^{+}host5	pAF50
b) pAF408 + pAF210	gDNA NEM316	pAF210	BamHI		pAF211
Construction of pTCVΩ*abx1* complementing vector					
pAF18 + pAF19	gDNA NEM316		EcoRI XbaI	pTCV-*erm*	
Construction of pTCVΩP_{cyl+}_*abx1* overexpression vector					
a) pAF52 + pAF53	gDNA CCH206		EcoRI	pTCV-*erm*	
b) pAF54 + pAF19	gDNA NEM316		XbaI		
Construction of pTCVΩP_{tet}_*abx1* overexpression vector					
a) pAF206 + pAF149	pTCV_P_{tet}	pAF206 +	EcoRI	pTCV-*erm*	
b) pAF150 + 1532_B	gDNA NEM316	1532_B	BamHI		
Construction of pTCVΩP_{cyl+}_*gbs1037* overexpression vector					
a) pAF52 + pAF203	gDNA CCH206	pAF52 +	EcoRI	pTCV-*erm*	
b) pAF204 + pAF205	gDNA NEM316	pAF205	BamHI		
Construction of pTCVΩP_{cyl+}_*EGFP* overexpression vector					
a) pAF52 + pAF200	gDNA CCH206	pAF52 +	EcoRI	pTCV-*erm*	
b) pAF201 + pAF202	EGFP	pAF202	BamHI		
Construction of pTCVΩ*abx1*_(Ala) alanine substitution vector					
a) pAF18 + X-rev	pTCVΩ*abx1*	pAF18 +	EcoRI	pTCV-*erm*	
b) X-fw + 1532_B	pTCVΩ*abx1*	1532_B	BamHI		
Construction of pTCVΩ*stp_stk* complementing vector					
pAF69 + pAF70	gDNA NEM316		XbaI	pTCV-*erm*	pAF174 pAF306

Construction of fusion proteins for double hybrid			
Cloning in pKNT25 and pUT18 (T18 / T25 tags at the C-terminal of the fusion proteins)			
Abx1 = pAF216 + pAF217	gDNA NEM316	HindIII EcoRI	pAF230
CovS = pAF218 + pAF219		PstI BamHI	pAF231
CovR = pAF220 + pAF221		HindIII EcoRI	pAF286
Gbs2082 = pAF239 + pAF240		PstI BamHI	
Gbs0430 = pAF245 + pAF246		PstI BamHI	
CovS_form I = pAF236 + pAF219		PstI BamHI	
CovS form II = pAF305 + pAF219		PstI BamHI	
CovS form III = pAF305 + pAF235		PstI BamHI	
Cloning in pKT25 and pUT18C (T18 / T25 tags at the N-terminal of the fusion proteins)			
Abx1 = pAF443 + pAF444	gDNA NEM316	BamHI KpnI	pAF208
CovS form IV = pAF500 + pAF517		BamHI KpnI	pAF209
CovS form V = pAF500 + pAF516		BamHI KpnI	pAF210
CovS form VI = (pAF500 + pAF501) + (pAF502 + pAF503)		BamHI KpnI	pAF211
CovS form VII = pAF500 + pAF503		BamHI KpnI	
